# Modified Near-Infrared Annealing Enabled Rapid and Homogeneous Crystallization of Perovskite Films for Efficient Solar Modules

**DOI:** 10.1007/s40820-025-01792-3

**Published:** 2025-05-22

**Authors:** Qing Chang, Peng He, Haosong Huang, Yingchen Peng, Xiao Han, Yang Shen, Jun Yin, Zhengjing Zhao, Ye Yang, Binghui Wu, Zhiguo Zhao, Jing Li, Nanfeng Zheng

**Affiliations:** 1 Pen-Tung Sah Institute of Micro-Nano Science and Technology and Fujian Key Laboratory of Semiconductor Materials and Applications, Xiamen University, Xiamen, 361005 People’s Republic of China; 2 Huaneng Clean Energy Research Institute, Beijing, 102209 People’s Republic of China; 3 College of Chemistry and Chemical Engineering, Xiamen University, Xiamen, 361005 People’s Republic of China; 4 Engineering Research Center of Micro-Nano Optoelectronic Materials and Devices, Ministry of Education, Xiamen University, Xiamen, 361005 People’s Republic of China; 5 Innovation Laboratory for Sciences and Technologies of Energy Materials of Fujian Province, Xiamen, 361005 People’s Republic of China

**Keywords:** Near-infrared annealing, Homogeneous crystallization, Blade coating, Perovskite solar modules

## Abstract

Currently, perovskite solar cells have achieved commendable progresses in power conversion efficiency (PCE) and operational stability. However, some conventional laboratory-scale fabrication methods become challenging when scaling up material syntheses or device production. Particularly, the prolonged high-temperature annealing process for the crystallization of perovskites requires a substantial amount of energy consumption and impact the modules’ throughput. Here, we report a modified near-infrared annealing (NIRA) process, which involves the excess PbI_2_ engineered crystallization, efficiently reduces the preparation time for perovskite active layer to within 20 s compared to dozens of min in conventional hot plate annealing (HPA) process. The study showed that the incorporated PbI_2_ promoted the consistent nucleation of the perovskite film, leading to the subsequent rapid and homogeneous crystallization at the NIRA stage. Thus, highly crystalized perovskite film was realized with even better crystallization performance than conventional HPA-based film. Ultimately, efficient perovskite solar modules of 36 and 100 cm^2^ were readily fabricated with the optimal PCEs of 22.03% and 20.18%, respectively. This study demonstrates, for the first time, the successful achievement of homogeneous and high-quality crystallization in large-area perovskite films through rapid NIRA processing. This approach not only significantly reduces energy consumption during production, but also substantially shortens the manufacturing cycle, paving a new path toward the commercial-scale application of perovskite solar modules.

**Supplementary Information:**

The online version contains supplementary material available at 10.1007/s40820-025-01792-3.

## Highlights

- Developing an infrared annealing system to achieve efficient annealing of perovskite films within 20 s.
- Fully blade-coated perovskite modules achieved remarkable efficiencies of 22.03% (6 × 6 cm^2^, active area: 18 cm^2^) and 20.18% (10 × 10 cm^2^, active area: 56 cm^2^)

**Supplementary Information:**

The online version contains supplementary material available at 10.1007/s40820-025-01792-3.

## Graphical abstract

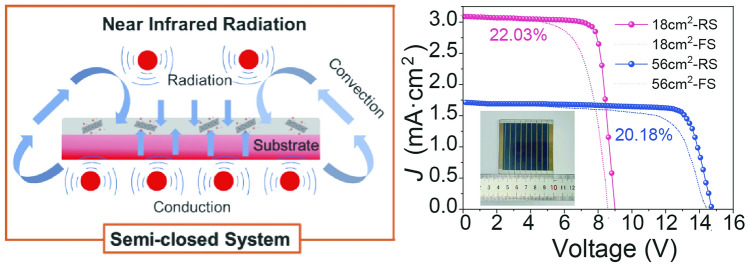

## Introduction

The lead halide perovskites have emerged as promising candidates for efficient and cost-effective photovoltaic material [1–3]. Currently, the commercialization of perovskite solar cells (PSCs) has gained significant momentum in the renewable energy sector [4, 5]. However, for large-area perovskite film fabrication, following the rapid solvent removal achieved through vacuum-flash evaporation or air-knife assisted techniques, thermal annealing must be precisely controlled for the final high-quality and uniform crystallization. The conventional thermal annealing process has drawn significant concern due to its time-consuming nature, expensive equipment requirements, and high energy consumption, which is the key bottleneck for large-scale preparation and throughput. Almost all high-performance PSCs with power conversion efficiencies exceeding 22% heavily rely on the conventional heat conduction method at temperatures > 100 °C for over 10 min. While, when scaling up the perovskite solar modules (PSMs) toward the industrialization, this process generally based on hot-plates become more and more difficult to implement for the controllable homogeneous crystallization besides energy and time consumptions [6, 7].

Reports have presented alternative techniques, such as microwave radiation annealing (MAP), intense pulse annealing (IAP) or near-infrared radiation annealing (NIRA), to facilitate the rapid preparation of perovskite thin films within seconds to minutes [8–10]. Among them, the NIRA annealing, typically utilizing near-infrared light with wavelengths ranging from 780 to 1400 nm, exhibits promising in enhancing the efficiency of energy transfer due to its simultaneous utilization of multiple heat transfer pathways including heat conduction, radiation and convection. This technology was pioneered by Watson et al. in 2015 to prepare a perovskite solar cell in an area of 0.1 cm^2^ with the efficiency of 10.7% by reducing the film annealing time to 2.5 s [11]. Subsequently, in 2019, Sandy Sanchez et al. further reduced the preparation time of perovskite films to 1.2 s through infrared fast heat treatment process combined with spin coating technology, and successfully prepared a perovskite single cell (aperture area of 0.09 cm^2^) with an efficiency of over 18% [12]. In the same year, Bosky Sharma et al. established a continuous heat treatment platform by using near-infrared radiation annealing technology and realized the production mode of roll-to-roll, which provided a new idea for industrialization in PSMs [13].

However, as the perovskite films are prepared from solution, commonly the precursors start to nucleate when the solvent reaches the supersaturation limit under vacuum-flash conditions, while then experience the rapid crystallization and phase transition during the NIRA annealing process due to the lack of the prolonged crystal growth (also called Ostwald ripening) [14, 15]. As a result, the quality of as-produced perovskite films cannot be guaranteed. Previous studies have demonstrated that obtaining a sufficient number of uniformly distributed nuclei and their subsequent close interconnection, during the nucleation stage of perovskite, is crucial for enhancing the crystalline quality of perovskite thin films [16, 17]. Notably, it has been reported that excess PbI_2_ can effectively regulate crystal quality, reduce *J-V* hysteresis, and increase the photostability [18, 19]. Therefore, when using NIRA thermal treatment, an appropriate strategy to balance nucleation and accelerate the crystallization process during perovskite film preparation is crucial for ensuring optimal crystal growth, especially in the context of large-scale commercial production [20].

In this study, a compositional engineering strategy together with the design and development of NIRA equipment was proposed and implemented to control the crystallization of perovskite films in large areas through the NIRA thermal treatments within 20 s. By optimizing the content of excess lead iodide (PbI_2_) in precursors to modify the homogeneous nucleation in the vacuum-flash stage and rapid crystallization during the NIRA, the growth of large-scale perovskite film with high crystallinity were well manipulated. Correspondingly, the 36 and 100 cm^2^ modules fabricated via NIRA achieved the remarkable efficiencies of 22.03% and 20.18%, respectively, thanks to the creation of breakthrough large-area films with exceptional quality. Furthermore, the large-area modules maintained 1000 h of* T*_90_ stability under ISOS-L-1 testing conditions. This insight offers a new perspective for cost reduction and efficiency enhancement in device manufacturing, holding significant potential for widespread applications in the field of optoelectronic devices.

## Experimental Section

### Materials

Cesium iodide (CsI), lead chloride (PbCl_2_), spiro-OMeTAD, PTAA, and FK-209 Co (III) TFSI salt were sourced from Xi’an Polymer Light Technology Corp. Lead iodide (PbI_2_) and formamidinium iodide (FAI) were purchased from TCI Chemicals. FcPF_6_ was obtained from Aladdin, while bis(trifluoromethane)sulfonimide lithium salt (Li-TFSI) was acquired from Alfa Aesar and Sigma-Aldrich. Chlorobenzene (CB, 99.9%) and N-methylpyrrolidone (NMP, 99.5%) were supplied by Alfa Aesar, and 4-tert-butylpyridine (t-BP) was purchased from Advanced Election Technology Co.

### Precursor Preparation and Devices Fabrications

#### Precursor Preparation

The FA_0.90_Cs_0.10_PbI_3_ solution was prepared from a precursor solution containing CsI (0.10 mmol), FAI (0.90 mmol), PbI_2_ (1.00 mmol), and dimethylformamide (DMF, 600 μL), with additives of MACl (20 mg), PbCl_2_ (10 mg) and N-methyl-2-pyrrolidone (NMP, 96 μL). For the excess X%-PbI_2_, an additional X × 1.0 mmol of PbI_2_ was added to the precursor solution and stirred for 2 h as the perovskite precursor. The hole transport layer solution contains 1 mL chlorobenzene, 90 mg spiro-OMeTAD, 4.0 mol% acetonitrile solution of Li-TFSI, 20 µL t-BP, and 1.0% mol FcPF_6_. The mixture was shaken and mixed for 10 min before use.

#### Devices Fabrications

The schematic diagram of the laser scribing technology used for series connection of PSMs is shown in Fig. S1. The FTO substrate was treated with ozone for 15 min, and a compact ZnTiO_3_ layer was deposited by spray pyrolysis at 450 °C. After cooling down to room temperature, SnO_2_ nanoparticles: H_2_O = 1:3 solution was further blade-coated as the electron transport layer [21]. FA_0.9_Cs_0.1_PbI_3_ was used as the perovskite material, and the perovskite film was fabricated through a blade coating and vacuum-flash processing. The entire procedure was conducted within a N_2_-filled glove box, with the temperature controlled at (20 ± 1) °C and the relative humidity stabilized maintained at RH (25 ± 1)%. In the blade coating process, the substrate with the electron transport layer was positioned under a fully automated blade coater with precisely adjusted height. For the 36-cm^2^ substrates, the coating parameters were set as follows: initial blade speed of 1.6 mm s^−1^, a 20-mm constant speed coating section, followed by a 40-mm variable speed section with an acceleration of 0.05 mm s^−2^. For the larger 100-cm^2^ substrates, the initial speed remained at 1.6 mm s^−1^, with a 33-mm constant speed section and a 67-mm variable speed section at the same acceleration rate. Immediately after blade coating, the wet films were transferred into a vacuum chamber where the pressure was rapidly reduced from atmospheric pressure to 10 Pa within 1 min, followed by a 10-s holding period at this vacuum level. Finally, the perovskite films were annealed using either NIRA or HPA. Then, the substrate was quickly transferred into the NIRA chamber, of which the output power was set at 4 KW m^−2^ with a treatment duration of 20 s. The control group adopted the HPA method was heated at 150 °C for 60 min.

Spiro-OMeTAD was used as the hole transport materials. Gold was used as counter electrode material and prepared by hot evaporation method with a thickness of 80 nm. Specific steps for the solar modules were additionally used as follows: P2 and P3 channels was fabricated using a laser scribing machine to realize the sub-cell interconnection.

#### Modules Encapsulation

The peripheral regions of the large-area perovskite films were trimmed using laser scribing to remove excess material. Subsequently, an encapsulant layer of polyisobutylene (PIB) and a cover glass were applied onto each module. Finally, the assembled device was placed in a vacuum hot-press machine to laminate the module with the cover glass, maintaining a temperature of 90 °C for 10 min during the pressing process (**Fig. S2**).

### Characterization

X-ray diffraction (XRD) patterns were obtained using a Thermo Scientific ARL EQUINOX 3500 X-ray diffractometer equipped with a Cu Kα radiation source. The optical absorptions of perovskite solutions were determined using a UV–vis spectrophotometer (LAMBDA 1050^+^). External quantum efficiency (EQE) spectra were recorded using an Enlitech QE-R measurement system. The morphology was observed with a field-emission scanning electron microscope (SEM, Zeiss GeminiSEM500). Photoluminescence (PL) mapping was conducted using a laser Raman microscope (RAMAN-11, Nanophoton). Time-resolved PL (TRPL) spectra were acquired with an Edinburgh Instruments FLS1000 spectrometer. X-ray photoelectron spectroscopy (XPS) and ultraviolet photoelectron spectroscopy (UPS) analyses were carried out using an Escalab Xi + spectrometer (Thermo Fisher Scientific). Current–voltage (J-V) characteristics of the devices were measured using a Keithley 2400 source measure unit equipped with a Class AAA solar simulator (450 W xenon lamp, Newport 94023A). The infrared thermal imageries were measured by HM-300X industrial thermal imager from Guangzhou sat infrared technology co. ltd. The incident photon-to-current efficiency (IPCE) was determined using an automated Newport 66,902 measurement system equipped with a 100-W xenon light source, a monochromator for wavelength selection. Data acquisition was performed in direct current (DC) mode. The maximum power point (MPP) tracking was performed on a Multi-Channels Solar Cells Stability Test System from Wuhan 91PVKSolar Technology Co. Ltd., China. For continuous operational stability measurements (ISOS-L-1 protocol), the photovoltaic modules were maintained in ambient air at 60% ± 10% relative humidity. The backside temperature was stabilized at 25 °C using a cooling plate. The modules were subjected to AM 1.5G equivalent illumination using a solar simulator LED array without UV filtering, while a constant bias voltage corresponding to the initial MPP voltage was applied. The test system automatically adjusted the bias voltage every 24 h to compensate for performance changes. A multi-channel maximum power point tracking (MPPT) system continuously monitored the module performance under persistent illumination, with active thermal management provided by a recirculating cooling system. The aging test under 85 °C and 85% RH was conducted in a solar cell reliability test system (K3600, McScience). The transient reflection (TR) spectroscopy setup is powered by a Ti: sapphire laser amplifier (PHAROS-20W), which can generate 1028 nm pulse train with a temporal pulse width of < 290 fs and with a repetition frequency of 500 kHz. A branch of the fundamental beam was introduced into an optical parametric amplifier (TP-OR-ORPHEUS-HP) to produce the monochromatic pump pulses with different wavelengths. The pump pulses were then chopped at the frequency of 4900 Hz by an optical chopper. The pump pulse energy was attenuated by neutral density filter wheels. Another branch of the 1028 nm beam was attenuated and focused into sapphire crystals to generate the broadband probe pulses in the visible and near-infrared regions (500–950 and 1190–1660 nm). The time delay between the pump and probe pulses was controlled by a motorized translation stage. The pump and probe pulses were focused on the same spot of the sample. The focal size of the pump was adjusted intentionally to be much greater than that of the probe so that the excitation density in the probing area was homogenous. The incident angle of the probe beam was 45 degrees. The reflected probe pulses were sent into to the visible or near-infrared spectrometers.

## Results and Discussion

### Uncovering the NIRA-Induced Peculiar Crystallization of Perovskite Films

**Fig. 1:**
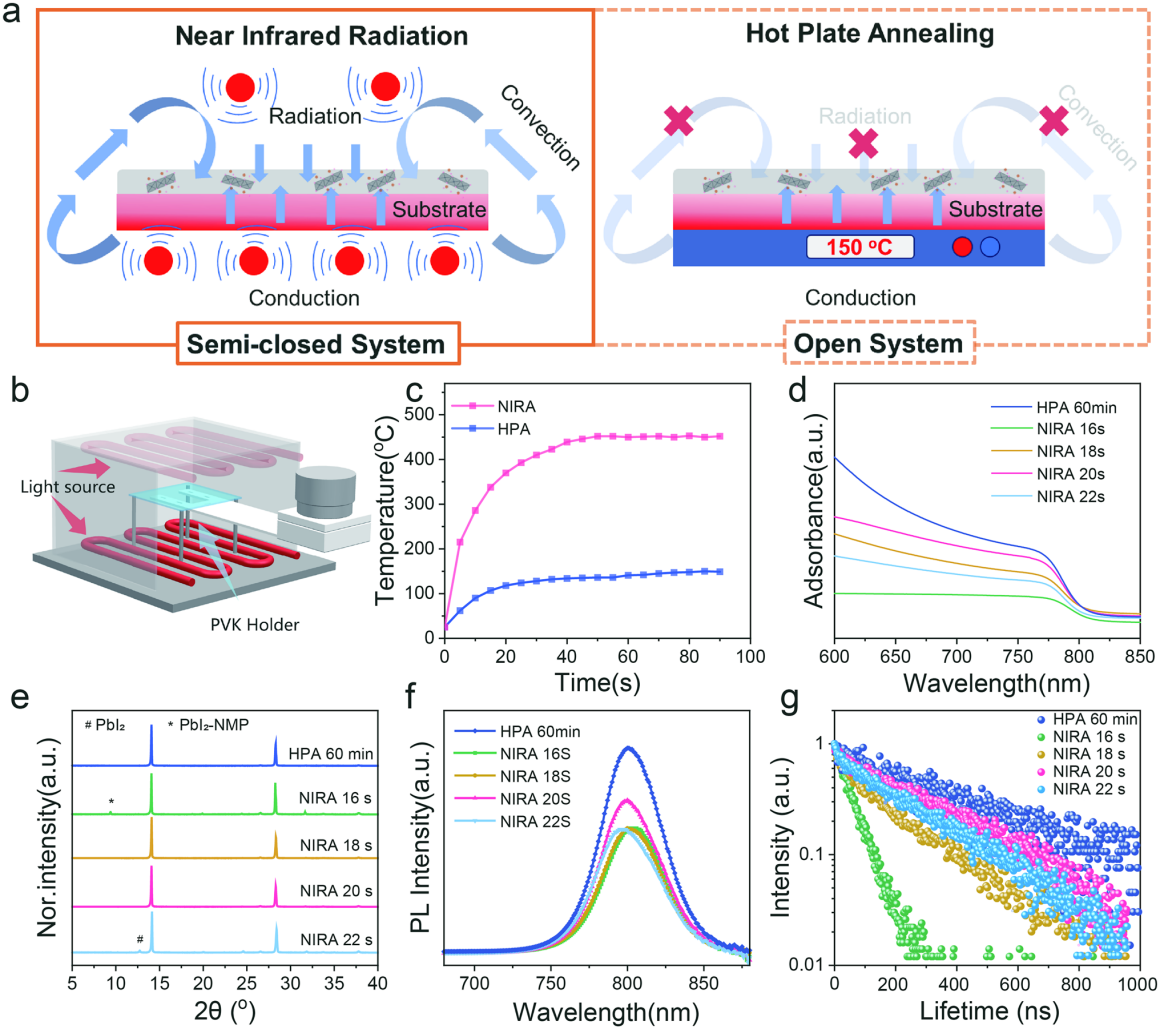
Feasibility examination on preparation of perovskite films by NIRA. **a** Schematic diagrams of heat transfer to perovskites based on NIRA and HPA methods. **b** Internal light source and structural diagram of NIRA equipment. **c** Temperature variation of NIRA and HPA processes (under 150 °C) as a function of time. **d** Uv–vis spectra, **e** X-ray diffraction (XRD) patterns, **f** Steady-state photoluminescence fluorescence intensities, and **g** the time-resolved photoluminescence (TRPL) spectra of NIRA and HPA annealed perovskite films

In order to verify the feasibility of NIRA in the preparation of perovskite films, the home-made NIRA equipment with uniform irradiation was designed and produced (as in **Fig. S3**), and then compared with the conventional HPA facility in heat employments. As illustrated in Fig. 1a, the NIRA facilitates the efficient concurrent thermal employment through the three kinds of energy transfer mechanisms: conduction, radiation, and convection [5, 22]. This approach mitigates the disadvantages associated with the single conduction mode of HPA, which can lead to uneven heat transfer, reduced efficiency, and energy wastage. While IR annealing demonstrates significant advantages for rapid large-area perovskite film fabrication, scaling up the film size introduces challenges in maintaining uniform thermal radiation, precise atmosphere control, and consistent temperature distribution across the substrate. To address this, our infrared chamber with reflective components and a semi-closed configuration could enhance heat distribution uniformity for mini-module fabrication (Fig. 1b). The modified NIRA system features an innovative semi-closed chamber design that incorporates a four-point edge support system to actively counteract temperature gradients. For the preparation of larger-sized perovskite films in industrial-scale production, it is undoubtedly possible to use the aforementioned strategy to achieve uniformity in annealing treatment and consistency in film crystallization across a large area. All the perovskite films’ preparations in this study were carried out by the blade coating process followed by vacuum-flash treatment. A series of heating durations (16, 18, 20, and 22 s) were used to anneal the perovskite films by NIRA. The samples were named as NIRA 16 s, NIRA 18 s, NIRA 20 s, and NIRA 22 s, respectively, and the films were compared with the conventional HPA for 60 min (the sample named as HPA 60 min). The Uv–vis spectra, XRD, steady-state PL and TRPL analyses were conducted to determine the optimal annealing time for the film under infrared treatment.

The irradiation spectrum of the NIR lamp is shown in **Fig. S4**. By monitoring the surface temperature of FTO under NIRA (measured by wire thermocouples), the heating curves of this heating method can be obtained, as shown in Fig. 1c**.** It can be found that up to 300 °C was rapidly achieved for 20 s when using the output power density of 4 KW m^−2^ of NIRA. The results demonstrated that the NIRA is theoretically feasible to provide enough energy for the crystallization or phase transition of perovskite films within a short time. The absorption spectra (Fig. 1d) indicated that the optimal annealing duration of NIRA was 20 s, and exhibits poorer crystallinity when compared with the conventional HPA, that is, weaker light absorption. The results were consistent with the XRD patterns and PL characterization results (Fig. 1e-g**)**. The crystallization evolution of the perovskite films can be well observed from the XRD patterns. Incomplete crystallization of the precursor film presented when the NIRA annealing time of 16 s, showing as the coexisted perovskite phases and PbI_2_-NMP complex (marked with *) with diffraction peak at 9.4° [23]. With increasing the annealing time to 22 s, the intermediate PbI_2_-NMP complex phase vanished while additional PbI_2_ phase (marked with ^#^) with diffraction peak at 12.7° becomes observable, attributed to the degradation of perovskite caused by prolonged irradiation and elevated temperatures [24]. The evolution of the films was consistent with the appearance and color for various near-infrared exposure time (**Fig. S5**). Further, the steady-state PL spectra as in Fig. 1f display the strongest emission at the wavelength of 805 nm in the NIRA 20 s film, while the blue shift in the NIRA 22 s film can be resolved, which is attributed to the presence of PbI_2_, although all the NIRA-films exhibit less intensities in emission than that of the HPA 60-min film. The TRPL lifetime graph exhibits a similar trend (Table S1). Therefore, the optimal NIRA duration was 20 s.

The preliminary results demonstrated that the direct application of NIRA is obviously not suitable for the preparation of high-quality perovskite films with the same quality as HPA. The surface morphology characterization indicated that the NIRA processed perovskite film has a reduced grain size film with obvious defects when compared with the film by HPA (**Fig. S6**). It also indicated that the conventional vacuum-flash processed film might not be suitable for direct NIRA considering its special thermal energy transfer mode. This is because the film just after vacuum-flash processing is mainly in an intermediate phase, and its insufficient nucleation sites makes it impossible for the film to quickly absorb infrared light in a short time, and then convert it into heat to complete crystallization [25, 26]. Most important, the uneven nucleation characteristics also result in the film being unable to crystallize uniformly within a short period of time, while the long-term NIRA would cause partial decomposition of the perovskite film. In this regard, excess PbI_2_ (with different concentrations of X = 5%, 10%, and 15%, marked as X-PbI_2_) was proposed and added to the perovskite precursor to promote the rapid homogeneous nucleation, increase the thermal energy absorption at the initial stage, and the subsequent fast crystallization within a few tens of seconds by NIRA.

### ***PbI***_***2***_***-modified Crystallization in NIRA for Improved Film Quality***

**Fig. 2:**
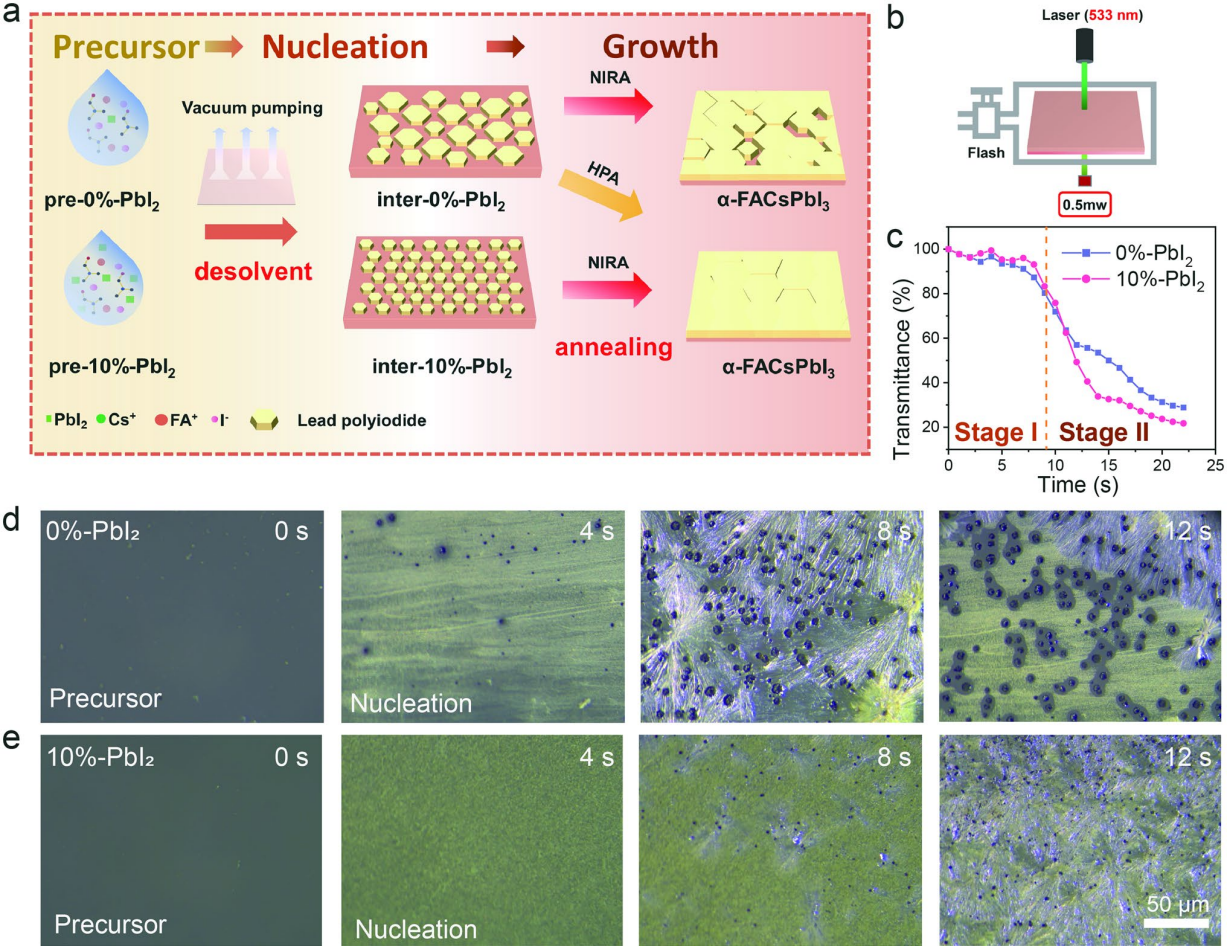
Nucleation and crystallization analysis of perovskite films. **a** Schematic illustration of the nucleation and crystallization process of perovskite films prepared from a 10%-PbI_2_ precursor solution under NIRA and HPA conditions. **b** Pattern of transmittance detection schematic and **c** normalized transmittance curve of in situ transmittance detection. Nucleation process of **d** 0%-PbI_2_ and **e** 10%-PbI_2_ perovskite wet films under optical microscope

Firstly, we conducted a screening to determine the optimal addition amount of PbI_2_. From the XRD patterns in **Fig. S7**, it can be observed that the diffraction intensity related to the perovskite films under NIRA enhances with the introduction of PbI_2_. Notably, the film grown with 10%-PbI_2_ addition exhibits superior crystallinity, while the utilization of 15%-PbI_2_ results in the emergence of a PbI_2_ diffraction peak at 12.6°. Further, the steady-state PL spectra shown in **Fig. S8** confirm that the film with 10%-PbI_2_ achieves a higher fluorescence intensity even compared with that in the HPA film, whereas the blue shift observed in the 15%-PbI_2_ film is attributable to the extra PbI_2_. In conclusion, together with the SEM morphology analysis shown in **Fig. S9**, an addition of 10%-PbI_2_ can be determined as the optimal concentration to grow high-quality perovskite films under NIRA conditions. Based on the optimal film quality of perovskite with 10%-PbI_2_, the manipulation effects of PbI_2_ on the nucleation and crystallization of perovskites under NIRA is proposed in Fig. 2a. Specifically, the precursors for 0%-PbI_2_ and 10%-PbI_2_ films have been designated as pre-0%-PbI_2_ and pre-10%-PbI_2_, respectively. For 0%-PbI_2_ precursor films, the nucleation process generally is spontaneous and the nucleation consistency is poor during the vacuum-flash process, which is not conducive to the rapid NIR absorption and crystallization of the film during the NIRA stage, resulting in pinholes and low crystallinity. When introducing the additional PbI_2_ in the precursor, the excess PbI_2_ would act as the initial nucleation point and promote the uniform nucleation during the vacuum-flash process, so that the grains of the film can be uniformly rapidly matured and crystallized during the NIRA process.

To reveal the effect of excess PbI_2_ on the nucleation and crystallization process of perovskite film, the in situ transmittance was firstly performed to monitor the transparency of the blade-coated films during the vacuum-flash treatment (Fig. 2b) [27]. Compared to the 0%-PbI_2_ sample (**Fig. S10)**, the 10%-PbI_2_ film exhibited accelerated transmittance over time. The normalized transmittance curves derived from *in situ* measurements are presented in Fig. 2c. Simultaneously, the nucleation performance of perovskite precursors with and without PbI_2_ modification during the vacuum-flash treatment was examined under the optical microscope in Fig. 2d, e. The results indicated that the 0%-PbI_2_ sample exhibits a continually emerged nuclei and distinct grain structures within 8 s. In contrast, the sample treated with 10%-PbI_2_ shows less nuclei and smaller and denser grains when within 8 s, but shows a noticeable enhanced rapid and homogeneous nucleation after 12 s. Therefore, it can be evidenced that the excess PbI_2_ indeed has modified the nucleation process, and the evolution was consistent with the transmittance of the film: At the first stage (Stage I) within 8 s, the nucleation has been suppressed for the 10%-PbI_2_ precursor film due to the stoichiometric imbalance induced by the additionally PbI_2_, exhibiting as the higher transmittance in comparison with the 0%-PbI_2_ film. At the second stage (Stage II) over 10 s, rapid and homogeneous nucleation occurs instantaneously by the assistance of excess PbI_2_ when the solvent has been basically evaporated by the vacuum-flash treatment. As a result, the transmittance of the sample decreases rapidly at this stage, which will be more conducive to the subsequent absorption of infrared light and crystallization.

**Fig. 3:**
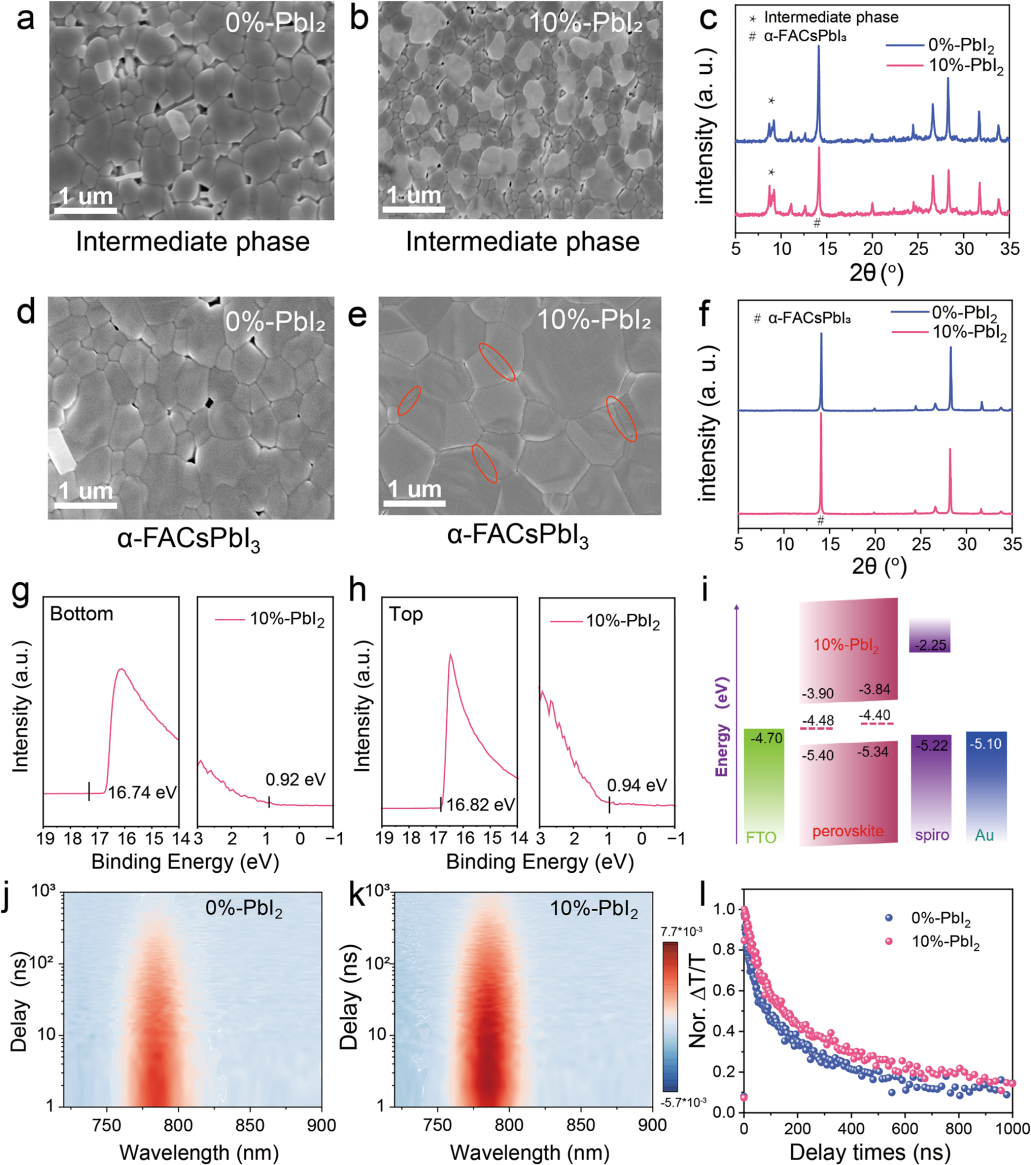
Growth and quality analysis of 10%-PbI_2_ perovskite films under NIRA and HPA conditions. SEM images of perovskite mesophase films of **a** 0%-PbI_2_ and **b**10%-PbI_2_ samples. SEM images of perovskite films of **d** 0%-PbI_2_ and **e** 10%-PbI_2_ samples after HAP and NIRA annealing processes. XRD pattern of 0%-PbI_2_ and 10%-PbI_2_ Perovskites films in **c** mesophase and **f** α-FACsPbI_3_.The UPS spectra of **g** Bottom and **h** Top perovskite films for 10%-PbI_2_ sample. **i** Schematic diagram of energy levels. TA spectra of the **j** 0%-PbI_2_ and **k** 10%-PbI_2_ perovskite films. **l** Normalized decay kinetics at 780 nm in TA spectroscopy of the 0%-PbI_2_ and 10%-PbI_2_ perovskite films

To verify the above hypothesis, we designated the vacuum-flash treatment perovskite films as the intermediate phase and the annealed-treated films as α-FACsPbI_3_. The temperature–time curve for NIRA-annealed films is shown in **Fig. S11.** Subsequently, we characterized both 0%-PbI_2_ and 10%-PbI_2_ samples through SEM and XRD measurements. Much uniform grains were observed from the 10%-PbI_2_ sample film, demonstrating the improved homogeneous nucleation, in comparison with the less-dense and nonuniform intermediate film morphology for the 0%-PbI_2_-based film (Fig. 3a, b). The higher intermediate phase diffraction and weaker perovskite peak shows good consistency with the morphology of the 10%-PbI_2_ sample film when compared with the 0%-PbI_2_-based film (Fig. 3c).

In comparison with the control film (0%-PbI_2_) showing numerous defects and prominent voids, the PbI_2_ engineered film (10%-PbI_2_) obtained a more compact and large crystalline structure, as shown in Fig. 3d, e. Apparently, the film underwent rapid Ostwald ripening during the NIRA process, which is manifested as a larger grain structure [28, 29]. The stronger diffraction peak at 14.8° compared to the 0%-PbI_2_ sample also demonstrated the higher crystallinity (Fig. 3f), which is consistent with the above surface SEM morphology. The AFM characterization (**Fig. S12**) reveals that the 10%-PbI_2_-modified perovskite film exhibits significantly reduced surface roughness (23 nm) compared to the 0%-PbI_2_ film (42 nm), along with markedly fewer pinholes, demonstrating improved film morphology. Additionally, the cross-sectional SEM images (**Fig. S13**) demonstrate that, under NIRA treatment, the 10%-PbI_2_ sample exhibits fewer defects at the grain boundaries and larger grain sizes compared to the 0%-PbI_2_ sample.

Notably, the utilization of optimal excess PbI_2_ was not detected by SEM and XRD, possibly due to the formation of an extremely thin PbI_2_ passivation layer that was below the resolution limit. The higher surface sensitive technique of XPS was employed to quantitatively analyze the I/Pb ratio (**Fig. S14** and **Table S2**). In the ABX_3_ structure of perovskites, the ideal stoichiometric ratio of I to Pb is 3:1, whereas in 10%-PbI_2_ excessed perovskite (AB_1+0.1_X_3+0.2_), the calculated ratio of I to Pb is 2.9:1. The trend of reduced I to Pb ratio at the surface is consistent with our estimation of a thin-layer PbI_2_ coverage on the perovskite surface. The UPS characterization was performed on both bottom and top surfaces of 10%-PbI_2_ perovskite films to investigate energy level alignment. As shown in Fig. 3g, h, the secondary electron cutoff region (left panel) and valence band region near the Fermi edge (right panel) demonstrate a Fermi level shift from 4.48 eV at the bottom surface to 4.80 eV at the top surface. This gradient energy level alignment, attributed to surface accumulation of PbI_2_ and its p-type doping effect, creates a favorable built-in electric field that facilitates efficient hole transport toward the top contact while suppressing charge recombination at interfaces. The surface-specific PbI_2_ passivation layer, while too thin to be detected by XRD or SEM, significantly modifies the interfacial energetics without compromising bulk optoelectronic properties. Thus, the energy levels for the perovskite related interface within the device can be schematically illustrated as in Fig. 3i. The modified Fermi level of the perovskite surface was further verified by Kelvin probe force microscopy, shown as the increased surface contact potential (SP), accompanied by the reduced surface roughness for the 10-PbI_2_ film (**Fig. S15**).

In order to verify the effect of the PbI_2_-induced interface on the charge transport performance of the device, the nanosecond transient absorption spectroscopy (ns-TA) was conducted to probe the charge carrier dynamics in the 10%-PbI_2_ NIRA processed perovskite film (10%-PbI_2_), in comparison with the 0%-PbI_2_ NIRA-treated film. The results were displayed in the pseudo-color images of the TR spectra (ΔT/T) of Fig. 3j, k. More information concerning measurements can be found in the Supplementary information section. A much slower decay rate in the 10%-PbI_2_ perovskite film than that of the 0%-PbI_2_ NIRA-treated film in Fig. 3i suggests the effective defects passivation in this film through PbI_2_, thereby increasing the carrier lifetime [30, 31]. Moreover, time-resolved terahertz (tr-THz) was conducted to compare the carrier mobility of 10%-PbI_2_ perovskite films. As shown in **Fig. S16a**, 10%-PbI_2_ perovskite films exhibit same THz transmission due to their similar preparation condition. In the pump-THz probe delay of 50 ps, 10%-PbI_2_ perovskite film exhibits much higher transition electronic field change than that in the 0%-PbI_2_-based film (**Fig. S16b**). At 50 ps after the excitation, the rate of terahertz electric field change (DE) of 10%-PbI_2_ perovskite film is larger than that of 0%-PbI_2_ sample (360.99 vs. 142.49 V/cm at 0 THz). These results indicate that PbI_2_ layer on the perovskite’s upper surface can passivate crystal defect ion and enhance the carrier mobility, which further improve the device efficiency.

### Application of NIRA for Efficient Perovskite Solar Modules

**Fig. 4:**
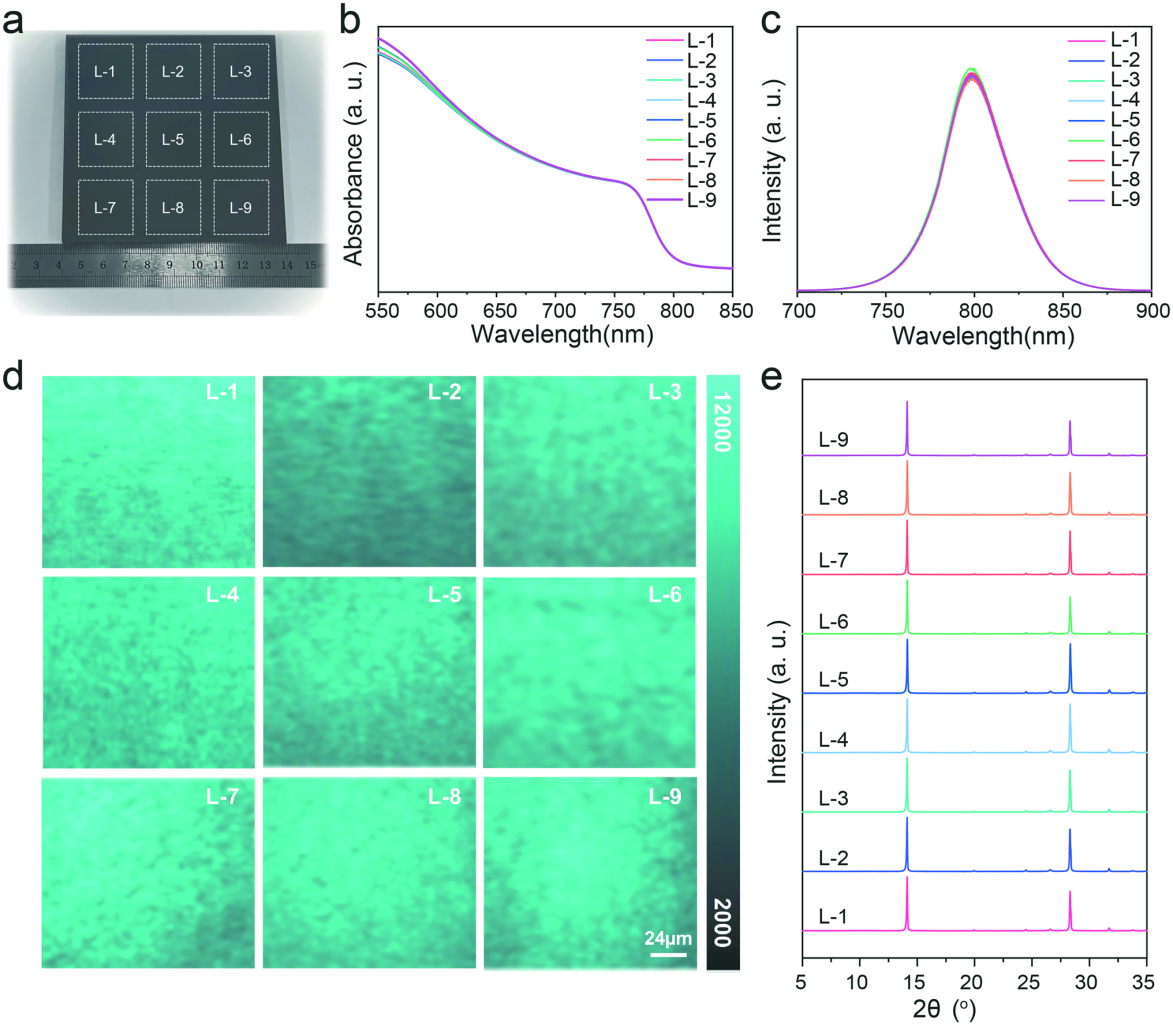
Uniformity verification of 10%-PbI_2_ perovskite films by NIRA.** a** Photograph of a 10 × 10 cm^2^ perovskite film. **b** Uv–vis spectra, **c** average steady-state photoluminescence fluorescence intensity, **d** the steady-state PL mapping and **e** XRD patterns of nine regions in perovskite films fabricated by NIRA process

To verify the application of NIRA in the preparation of large-area perovskite films and high-efficiency modules, the 10 × 10 cm^2^ size perovskite film was fabricated and the uniformity was evaluated by the characterization of nine equal sections, as shown in Fig. 4a. The UV–Vis spectra of the perovskite films in Fig. 4b indicated a consistent cutoff wavelength in terms of absorbance. The steady-state PL (Fig. 4c) demonstrates that the fluorescence intensity across the whole surface is almost identical, which can be more intuitively observed from the PL mapping images in Fig. 4d. The slightly dim luminescence observed at locations L-2 may be attributed to the accumulated liquid residue during the blade coating process. As a contrast, besides the region L-2, an even darker region at L-5 within the HPA film’s center in **Fig. S17** can be resolved, which is likely due to uneven crystallization during HPA, where the crystal growth from the edges toward the center results in increased stress at the film’s central position, leading to poorer crystallization in that area. The SEM images of the NIRA-treated perovskite films reveal uniform morphology across different regions (**Fig. S18**), with consistent grain sizes, confirming homogeneous crystallization induced by NIRA [32, 33]. And the results were consistent with the recorded XRD patterns from the nine areas (Fig. 4e). Obviously, the PbI_2_-modified novel nucleation and rapid crystallization by NIRA is more conducive to the preparation of higher quality large-area perovskite films.

**Fig. 5:**
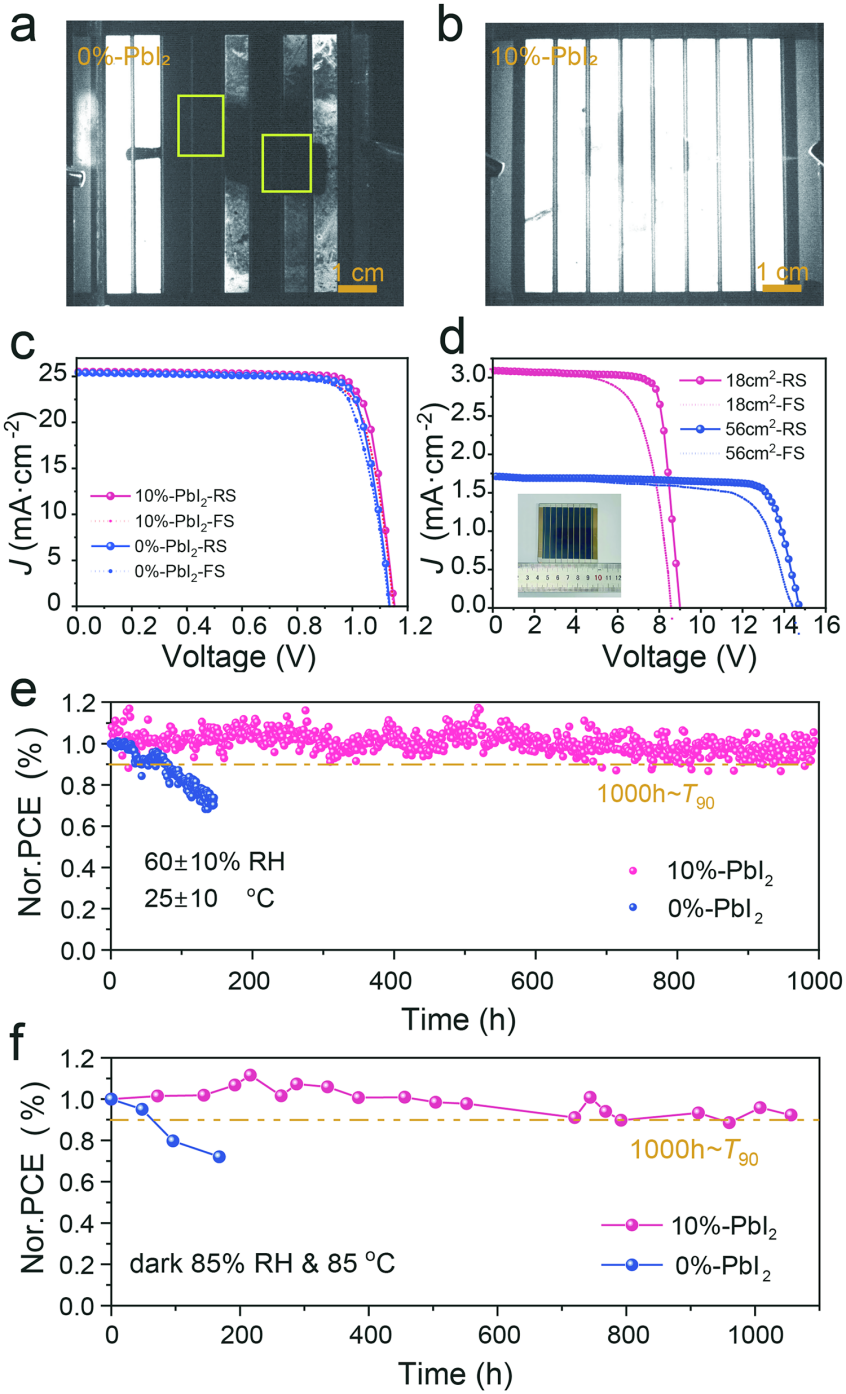
Performance and stability of NIRA-PSMs. **a** 0%-PbI_2_ and **b** 10%-PbI_2_ PSC module electroluminescence (EL) images. **c** Champion PCE of small-area perovskite solar cells. **d** Reverse scanned *J–V* curves of the 6 × 6 cm^2^ and 10 × 10 cm^2^ 10%-PbI_2_ devices based on NIRA, with the cross-sectional SEM image in the inset. **e** Operational stability of the 0%-PbI_2_ and 10%-PbI_2_ PSCs under MPPT, AM 1.5 G, 100 mW cm^−2^ continuous illumination in an air atmosphere. **f** Storage stabilities of encapsulated PTAA-based 0%-PbI_2_ and 10%-PbI_2_ devices at 85 °C in 85%-RH air

The n-i-p PSMs in size of 6 × 6 and 10 × 10 cm^2^ with device structure of FTO/ZTS/SnO_2_/Perovskite/Spiro-OMeTAD/Au were fabricated and tested under simulated 1 sun illumination at an intensity of 100 mW cm^−2^ (AM 1.5 spectrum) [21, 34]. The fabricated modules exhibit a geometric fill factor (GFF) of 95.60% as shown in **Fig. S19**. The bright and uniform electroluminescence (EL) image of Fig. 5a, b demonstrated that the fabricated 10%-PbI_2_ PSMs has a remarkable uniform carrier transporting and device working states, which should be benefited from the homogeneous and highly crystalized perovskite film through the PbI_2_ engineered NIRA [35, 36]. The result is also consistent with the subsequent infrared thermal imaging (**Fig. S20**) when the module driven under a reverse bias, showing as the relative uniform without resolvable hot spots [37]. Based on the uniform crystallization of high-quality perovskite films by PbI_2_ engineering in the NIRA, a notable photovoltaic conversion efficiency of 24.33% was attained in the typical small-area devices (0.12 cm^2^) as shown in Fig. 5c (**Table S3**) [38, 39]. The integrated current densities from IPCE spectra **(Fig. S21**) are 23.55 and 24.17 mA cm^−2^ for 0%-PbI_2_ and 10%-PbI_2_ devices, respectively, which agree well with the Jsc values obtained from *J-V* measurements (within ~ 5% deviation). As shown in **Fig. S22a**, the 10%-PbI_2_ device exhibited a significantly lower dark current compared to the 0%-PbI_2_ device under dark conditions. The defect density of the perovskite films was quantitatively assessed using space charge-limited current (SCLC) analysis (**Fig. S22b**). The trap-filled limit voltages (*V*_TFL_) derived from the dark condition *J-V* curves were recorded at 0.85 and 0.62 V for the 0%-PbI_2_ and 10%-PbI_2_ devices, respectively, corresponding to trap densities of 1.15 × 10^16^ and 8.39 × 10^15^ cm^−3^. This reduction in defect density is attributed to the improved crystallinity of the perovskite, which leads to fewer defects.

The champion PCE for the PSMs with areas of 36 cm^2^ (active area 18 cm^2^) and 100 cm^2^ (active area 56 cm^2^) achieved 22.03% and 20.18%, respectively (Fig. 5d, **Table S4**). The achievement of the 10%-PbI_2_ NIRA-treated modules has demonstrated outstanding performance advantages in current blade-coated PSMs as summarized in **Fig. S23** and **Table S5**. The observed hysteresis in the devices primarily stems from the absence of additional surface passivation, an experimental strategy designed to unambiguously elucidate the specific role of 10%-PbI_2_ in modulating perovskite crystallization dynamics and device stability without confounding effects. This approach also establishes a foundation for future exploration of diverse passivation strategies. Additionally, the *J-V* curves of perovskite modules fabricated with HPA treatment compared to those with 0%-PbI_2_, 5%-PbI_2_, and 15%-PbI_2_ NIRA treatment are presented in **Fig. S24** (**Table S6**), demonstrating the superior performance of 10%-PbI_2_ modules under optimized NIRA processing. It is evident that our NIRA technique can achieve one of the highest photovoltaic conversion efficiencies for the blade-coated perovskite films. The operational stability of the encapsulated PSMs at the maximum power point (MPP) (25 ± 10°C, RH 60 ± 10%) is further evaluated as shown in Fig. 5e and **Table S7**. Under the continuous AM 1.5 G illumination, it was found that the module based on the NIRA-treated film with 10%-PbI_2_ could maintain 90% of initial PCE (*T*_90_) for 1000 h. Simultaneously, the hydrothermal stability was tracked in an aging oven with 85% RH at 85 °C, as shown in Fig. 5f and **Table S8**, the encapsulated PSMs maintained 91% (14.56%) of the initial efficiency after 1000h, proving that the 10%-PbI_2_-based device has good light and thermal stability, which is superior than the 0%-PbI_2_-based devices.

## Conclusions

In summary, a novel thermal treatment technique of NIRA together with excess PbI_2_ engineering promoted crystallization was proposed and demonstrated for producing large-area high-quality perovskite films and corresponding solar modules. This method uses a collaborative mechanism of modified nucleation, improved IR absorption and accelerated homogeneous crystallization, so that achieving a rapid film growth within a short timeframe of dozens of seconds (e.g., 20 s). The introduced excess PbI_2_ was found to be cleverly distributed on the surface of perovskite grains after NIRA treatment, further acting as an ideal passivation layer to improve the charges’ transport performance of the devices. As a result, champion PCEs of PSMs in the areas of 36 cm^2^ (active area 18 cm^2^) and 100 cm^2^ (active area 56 cm^2^) were readily achieved by 22.03 and 20.18%, respectively. The NIRA process PSMs also exhibited *T*_90_ stability under the ISOS-L-1 standard and preserved *T*_91_ stability under accelerated aging conditions of 85% humidity and 85 °C for 1000 h. This work provides a universal strategy for preparing high-quality perovskite films using the attractive infrared thermal annealing, showing great application prospects in the commercialization of large-area perovskite photovoltaics.

## Supplementary Information

Below is the link to the electronic supplementary material.

Supplementary file1 (DOCX 7144 kb)
