# Supplementary material for: Modified Near-Infrared Annealing Enabled Rapid and Homogeneous Crystallization of Perovskite Films for Efficient Solar Modules
Source: Nanomicro Lett. 2025 May 22;17:272. doi: 10.1007/s40820-025-01792-3 (PMC12098230; doi:10.1007/s40820-025-01792-3)
Supplement: Supplementary file 1 — Supplementary file1 (DOCX 7144 kb) [file 40820_2025_1792_MOESM1_ESM.docx]

Supporting Information for

**Modified Near-Infrared Annealing Enabled Rapid and** **Homogeneous Crystallization of Perovskite Films for Efficient Solar Modules**

Qing Chang ^1,^^4,5#^, Peng He ^3,5#^, Haosong Huang ^3,5^, Yingchen Peng ^3,5^, Xiao Han ^1,4,5^, Yang Shen ^3,5^, Jun Yin ^1,4,5^*, Zhengjing Zhao ^2^, Ye Yang ^3,5^, Binghui Wu ^1,5^, Zhiguo Zhao ^2^*, Jing Li ^1,4,5^*, and Nanfeng Zheng^3,5^

^1^ Pen-Tung Sah Institute of Micro-Nano Science and Technology and Fujian Key Laboratory of Semiconductor Materials and Applications, Xiamen University, Xiamen 361005, P. R. China

^2^ Huaneng Clean Energy Research Institute, Beijing 102209, P. R. China

^3^ College of Chemistry and Chemical Engineering, Xiamen University, Xiamen, 361005, P. R. China

^4^ Engineering Research Center of Micro-nano Optoelectronic Materials and Devices, Ministry of Education, Xiamen University, Xiamen 361005, P. R. China

^5^ Innovation Laboratory for Sciences and Technologies of Energy Materials of Fujian Province, Xiamen 361005, P. R. China

^#^Qing Chang and Peng He contributed equally to this work.

*Corresponding authors. E-mail: [lijing@xmu.edu.cn](mailto:lijing@xmu.edu.cn) (Jing Li); [jyin@xmu.edu.cn](mailto:jyin@xmu.edu.cn) (Jun Yin); [zg_zhao@qny.chng.com.cn](mailto:zg_zhao@qny.chng.com.cn) (Zhiguo Zhao)

**Supplementary Figures**


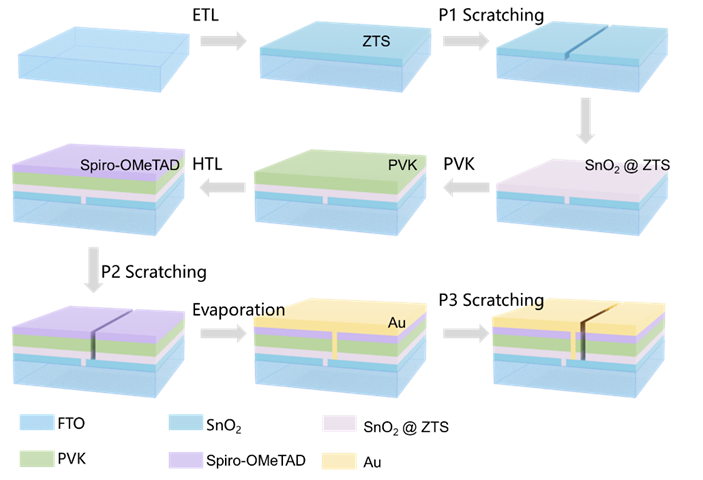


**Fig. S1** A schematic diagram of the laser scribing technology used for series connection of perovskite solar modules


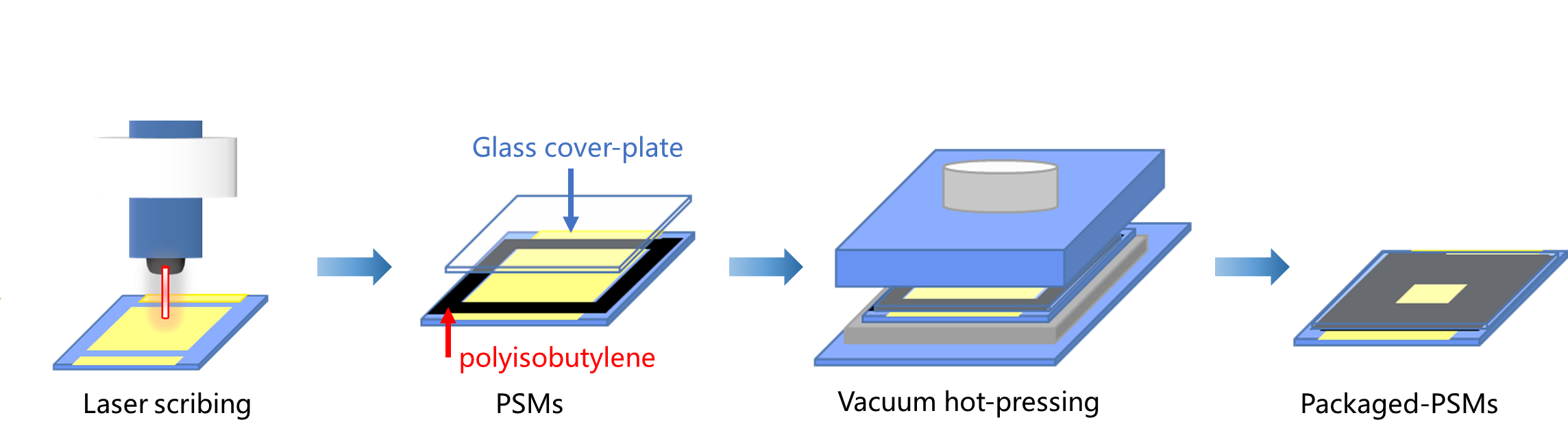


**Fig. S2** A schematic diagram of module encapsulation


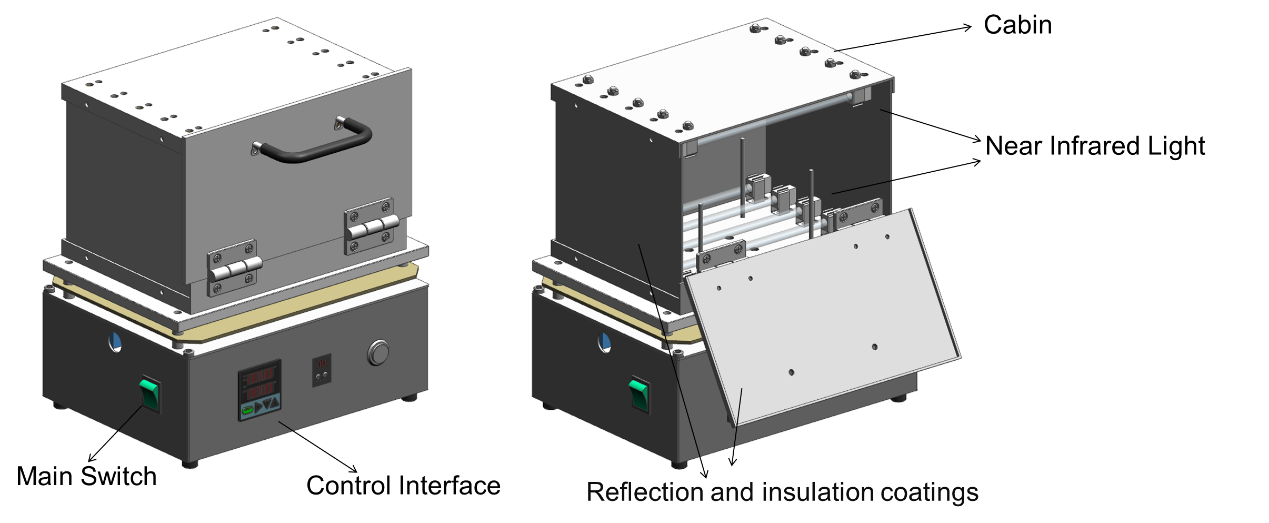


**Fig. S3** The construction of NIRA equipment


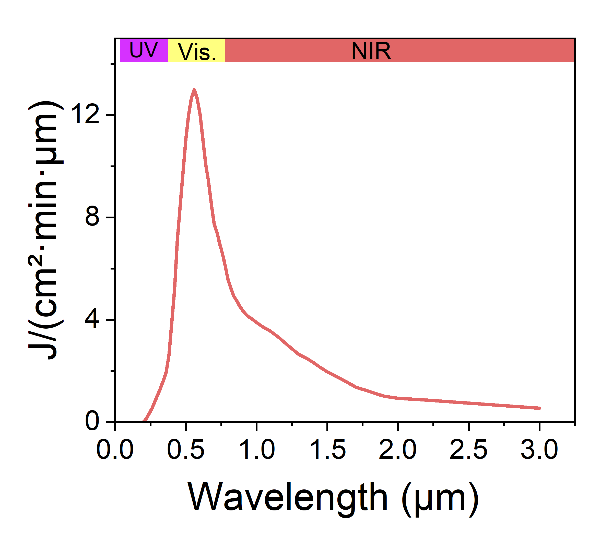


**Fig. S4** The irradiation spectrum of the NIR lamp


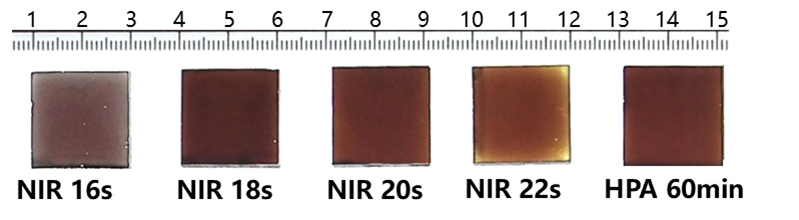


**Fig. S5** Photos of NIRA-annealed films for different annealing time compared to the hot plate annealed (HPA) film for 60 min


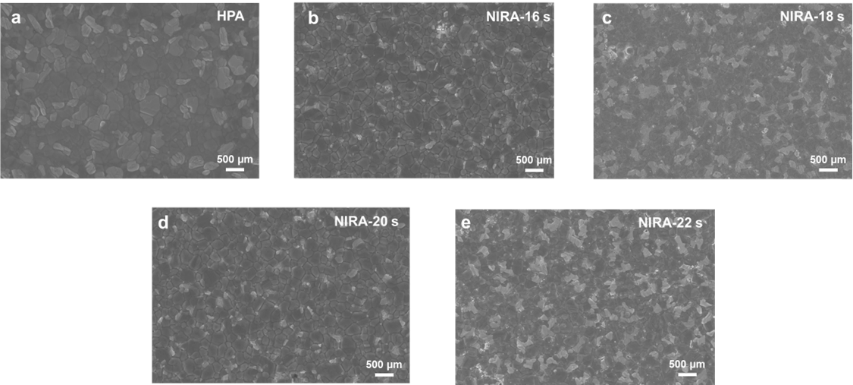


**Fig. S6** SEM images of perovskite films of (**a**) HPA, (**b**) NIRA-16 s, (**c**) NIRA-18 s, (**d**) NIRA-20 s and (**e**) NIRA- 22 s

**
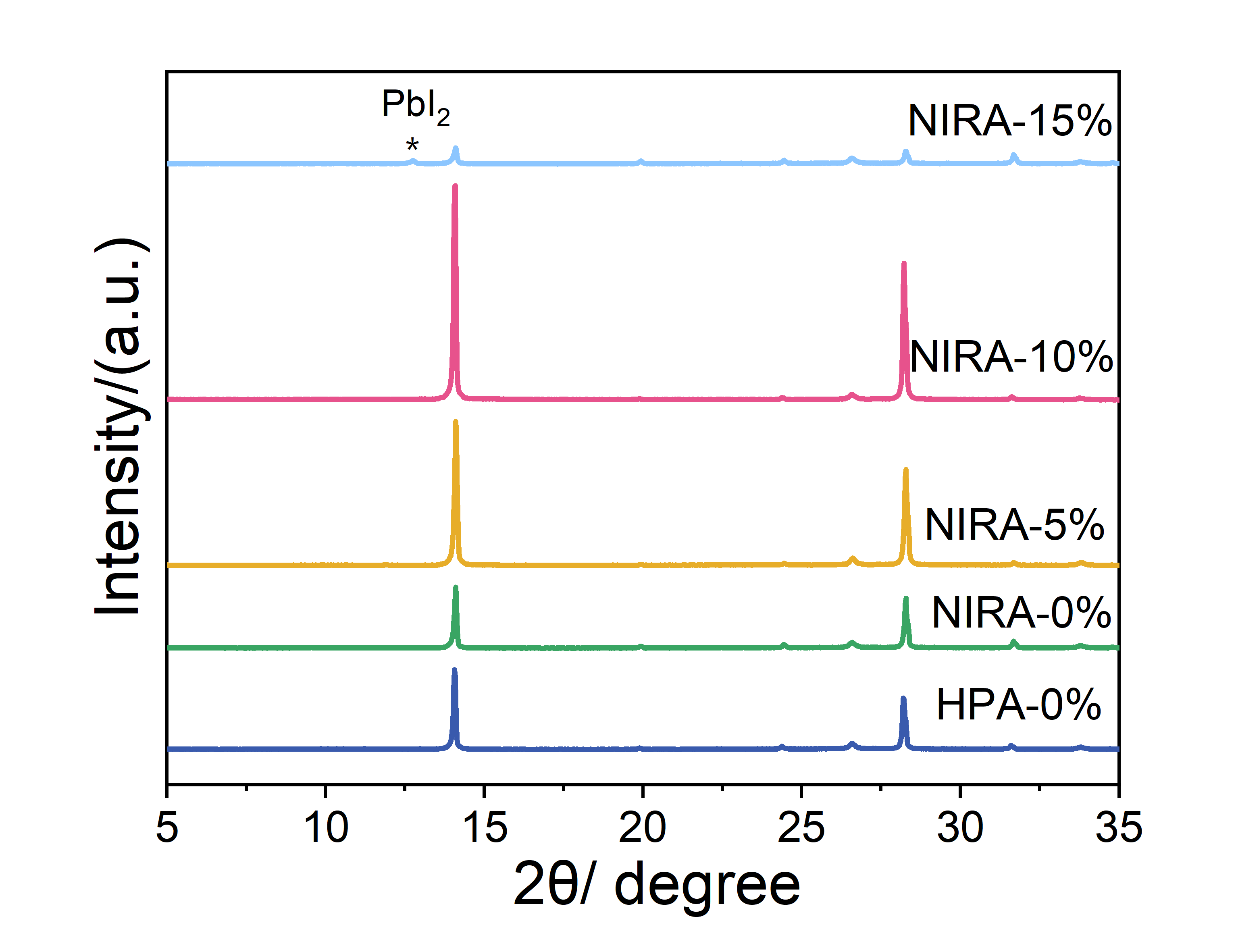
**

**Fig. S7** XRD patterns of perovskite films under HPA and under NIRA with different amount of excess-PbI_2_ (0%, 5%, 10%, and 15%) in precursors

**Fig. S8** Steady-state photoluminescence spectra (PL) of perovskite film under HPA and under NIRA with different amount of excess-PbI_2_ (0%, 5%, 10%, and 15%) in precursors

**
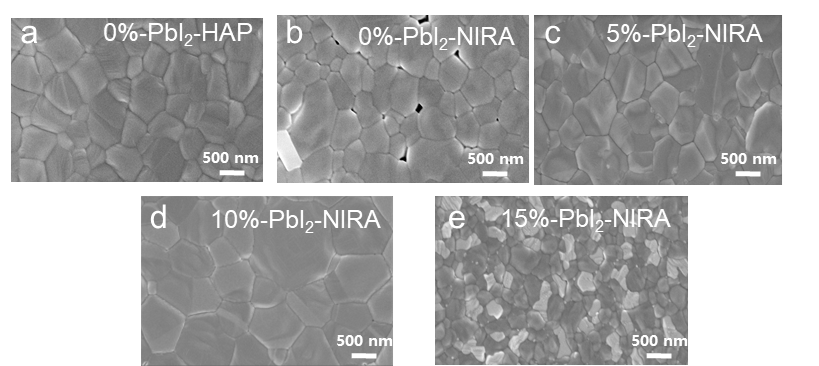
**

**Fig. S9** SEM images of perovskite films under HPA and under NIRA with different amount of excess-PbI_2_ (0%, 5%, 10%, and 15%) in precursors

**
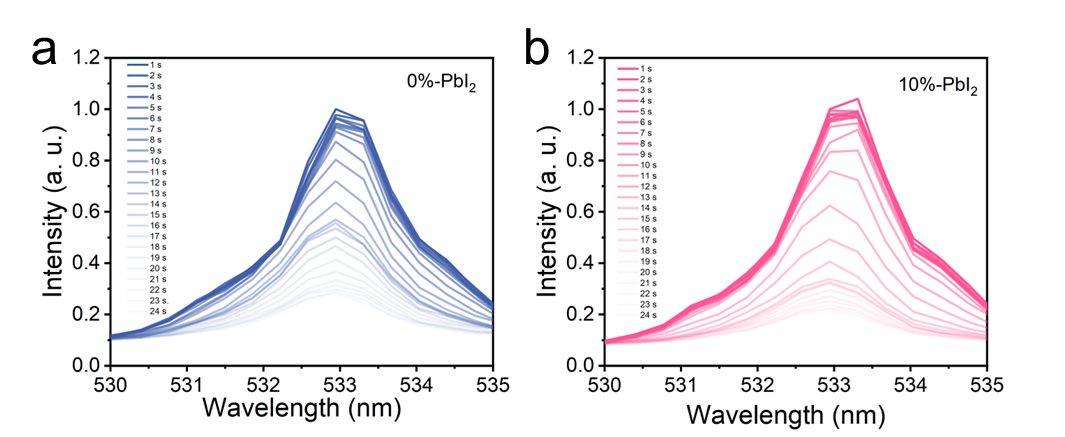
**

**Fig. S10** The transmittance spectra of (**a**) 0%-PbI_2_ and (**b**) 10%-PbI_2_ perovskite films during vacuum flash process

**
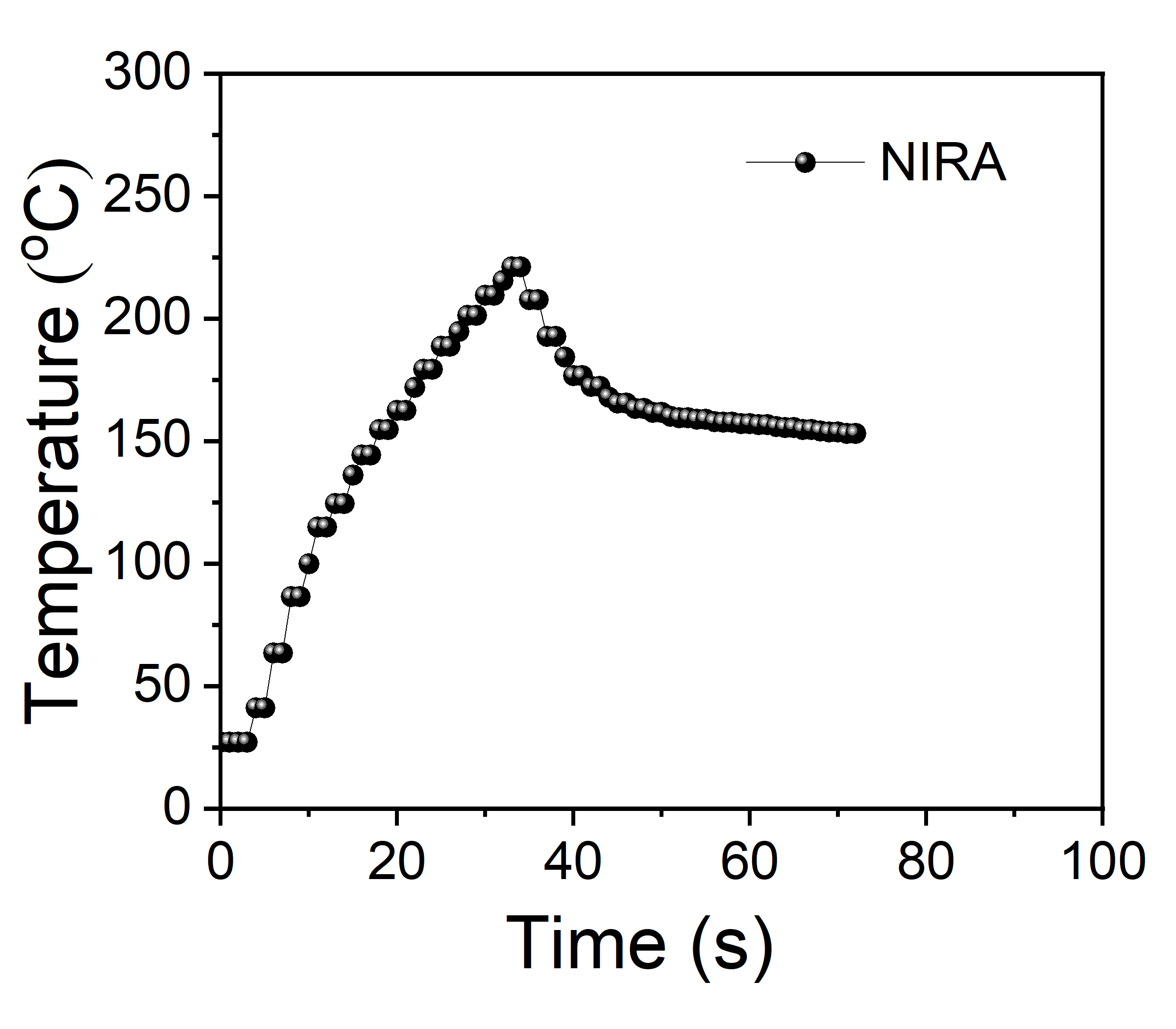
**

**Fig. S11** Temperature variation for FTO under NIRA as a function of time


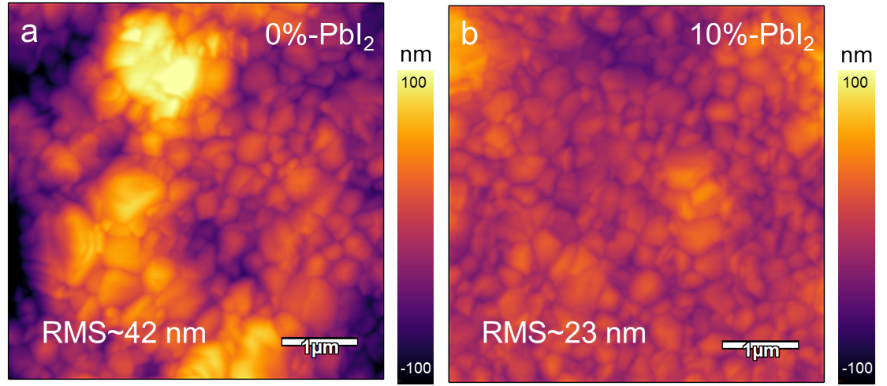


**Fig. S12** Atomic Force Microscopy (AFM) images of the (a) 0%-PbI_2_ and (b)10%-PbI_2_ modified perovskite films


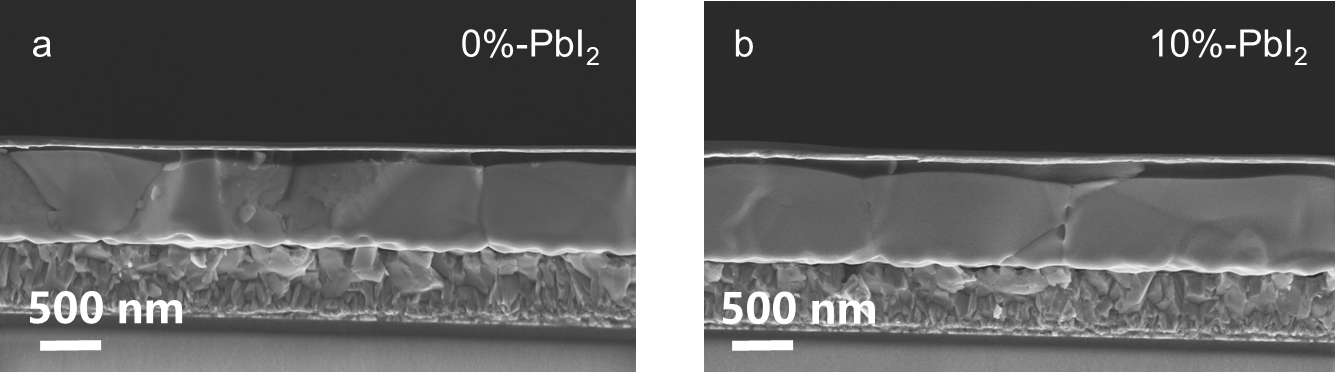


**Fig. S13** The cross-sectional SEM images of (**a**) 0%-PbI_2_ and (**b**) 10%-PbI_2_ perovskite films


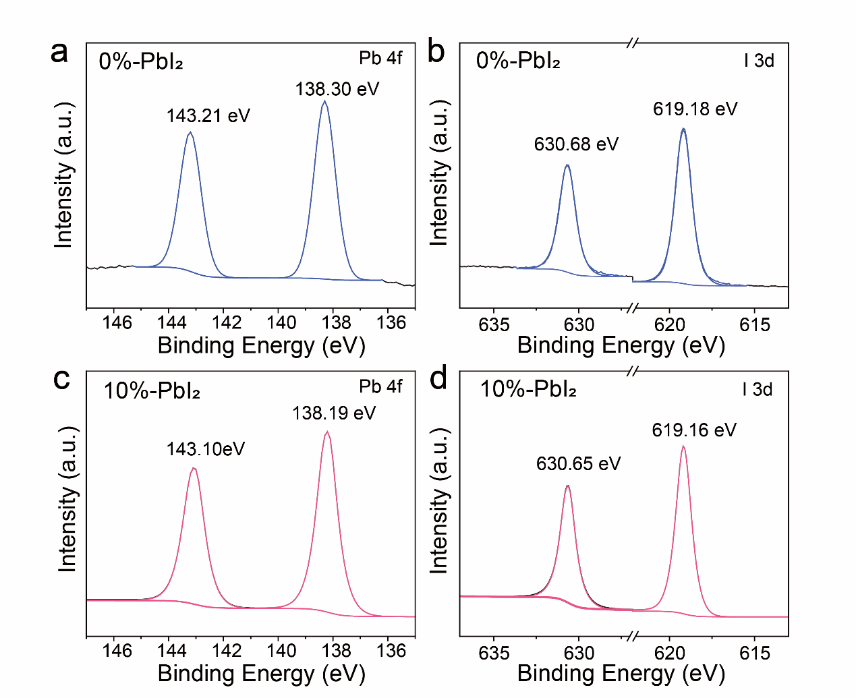


**Fig. S14** Pb 4f and I 3d signals in XPS spectra: (a, b) 0%-PbI_2_ and (c, d)10%-PbI_2_ perovskite films


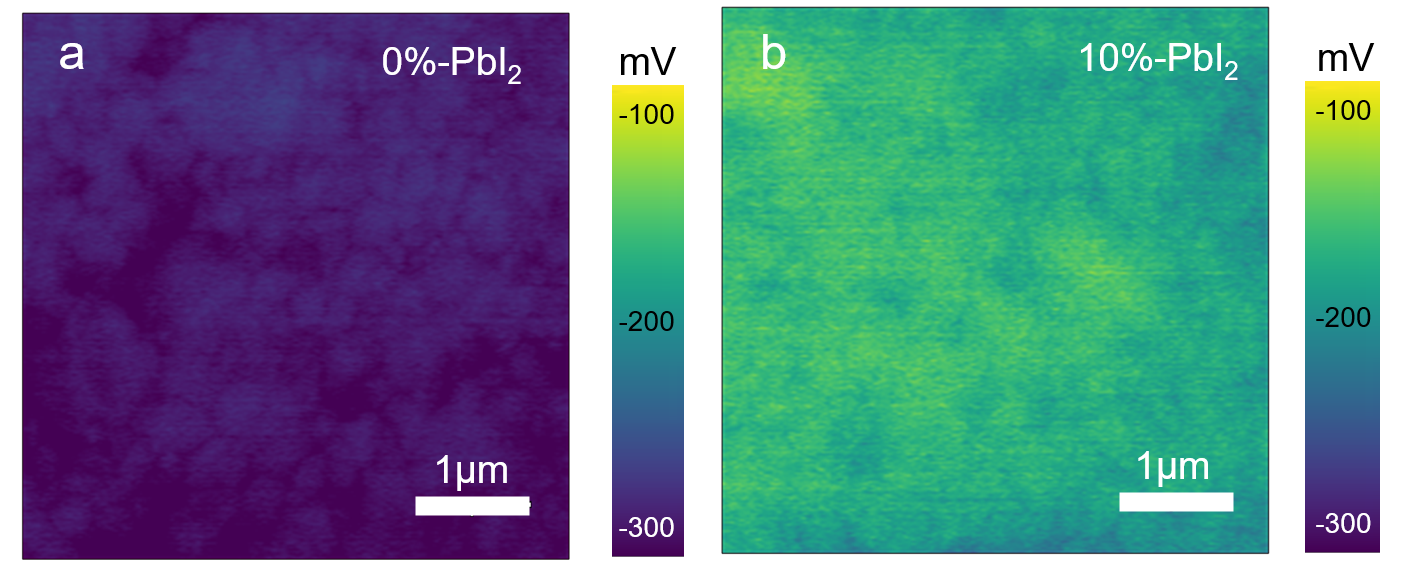


**Fig. S15** (**a**) 0%-PbI_2_ and (**b**) 10%-PbI_2_ perovskite films measured by kelvin probe force microscopy


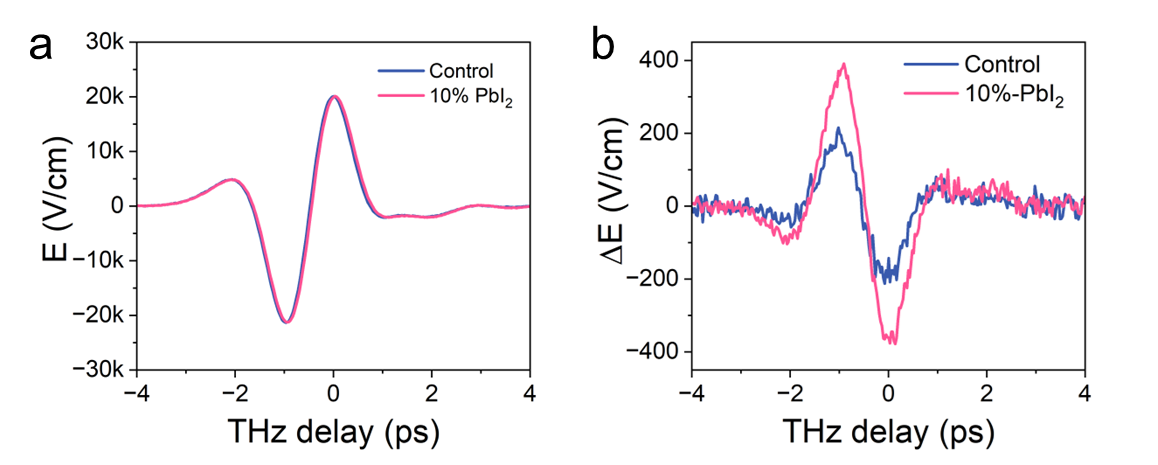


**Fig. S16** (**a**) Terahertz transmission spectra of 0%-PbI_2_ and 10%-PbI_2_ perovskite films. (**b**) Transient terahertz spectra change of 0%-PbI_2_ and 10%-PbI_2_ perovskite films at 50 ps pump-terahertz probe delay


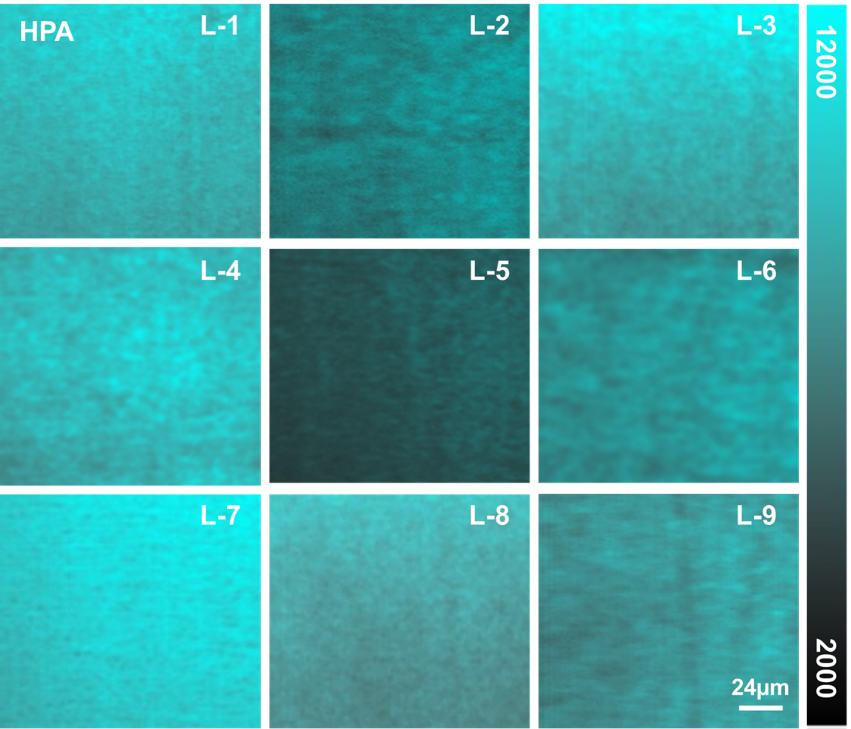


**Fig. S17** The steady-state PL mapping of nine regions in the perovskite film fabricated by HPA process


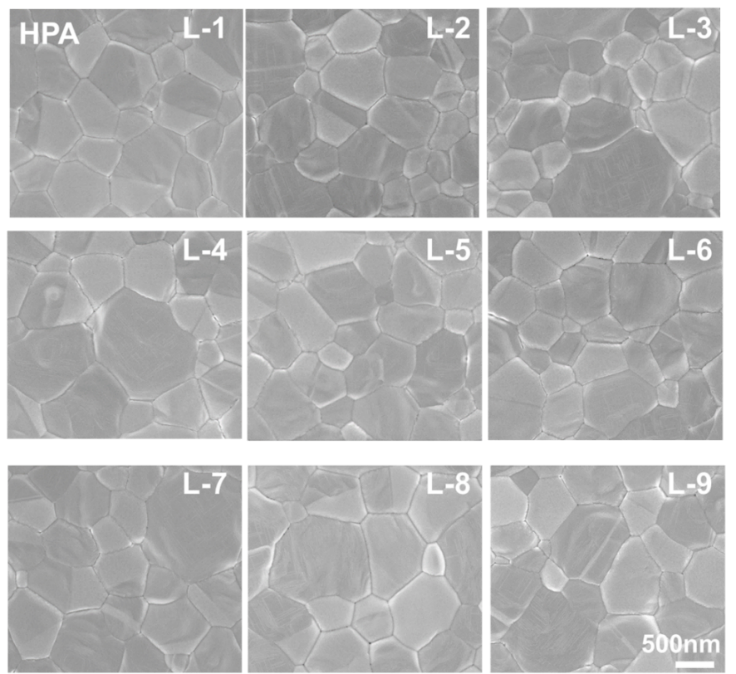


**Fig. S18** SEM images of nine regions in the perovskite film fabricated by HPA process


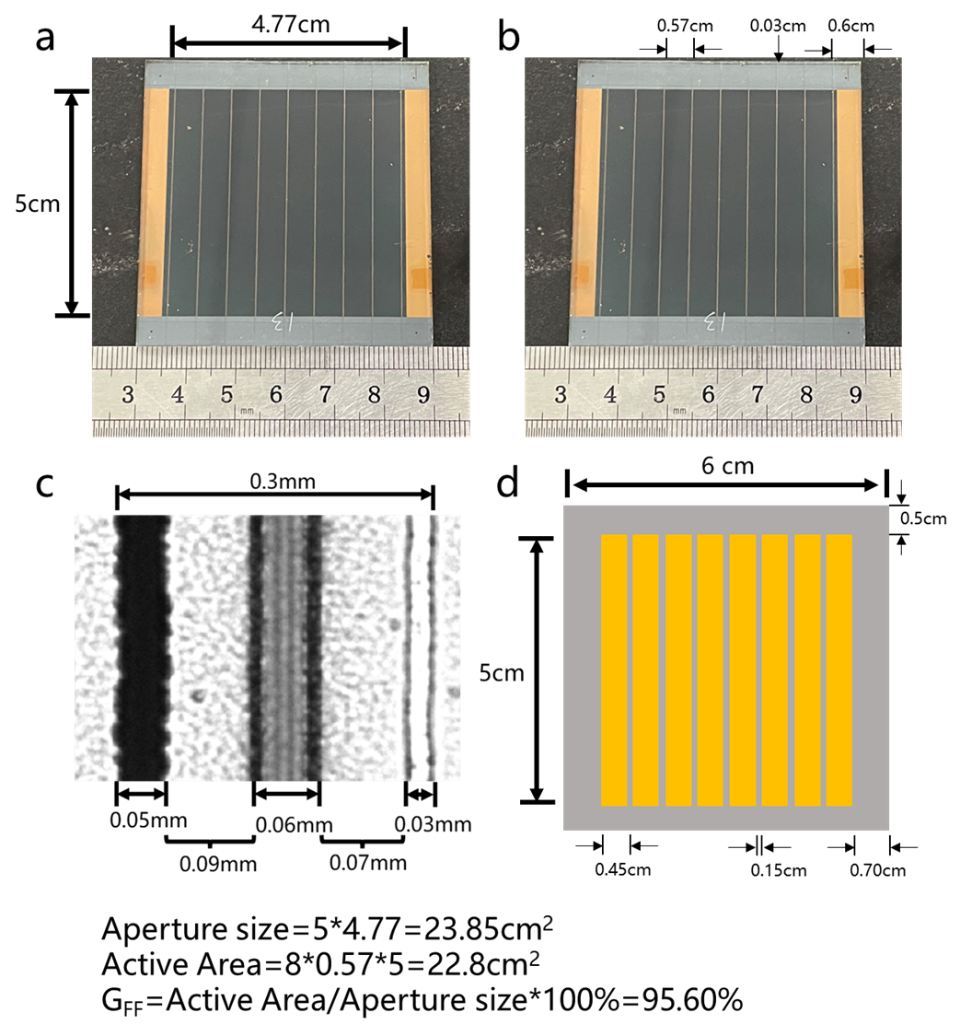


**Fig. S19** (**a**) aperture size, (**b**) sub-cell parameter and the definition of the active area, as well as geometric fill factor (GFF) for the typical 6 cm × 6 cm module. (**c**) Optical microscope (OM) photo shows the sub-cell's separation in the module and the related widths for P1, P2, and P3 lines. The calculations of aperture size, active area and the GFF was shown in the Figure. (**d**) the mask of 18 cm^2^ active area


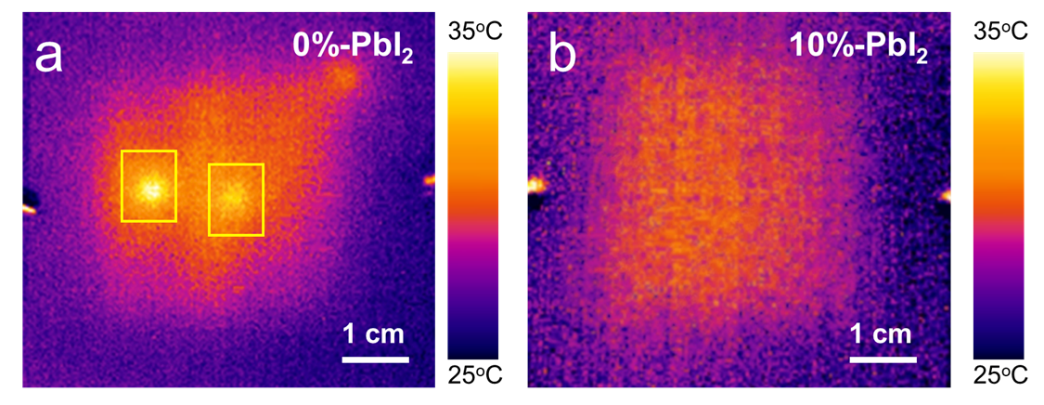


**Fig. S20**The infrared thermal imagery of PSMs at 6 V bias: (**a**) 0%-PbI_2_ and (**b**) 10%-PbI_2_


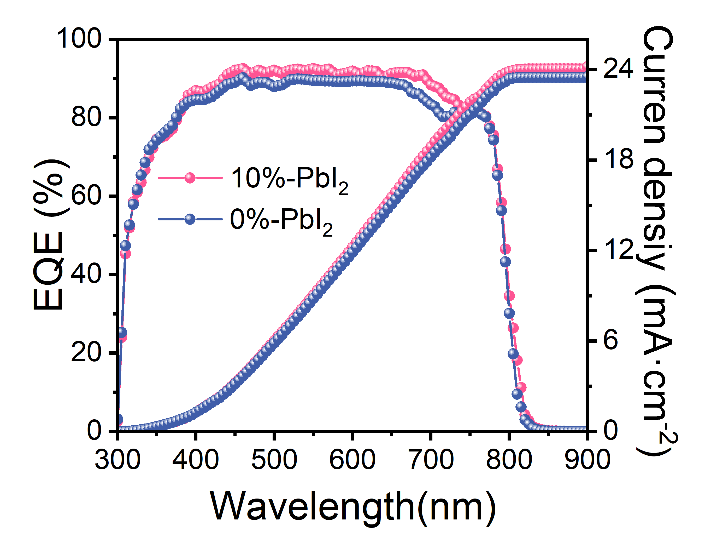


**Fig. S21** IPCE spectra and their integrated current densities for the 0%-PbI_2_ and 10%-PbI_2_ devices


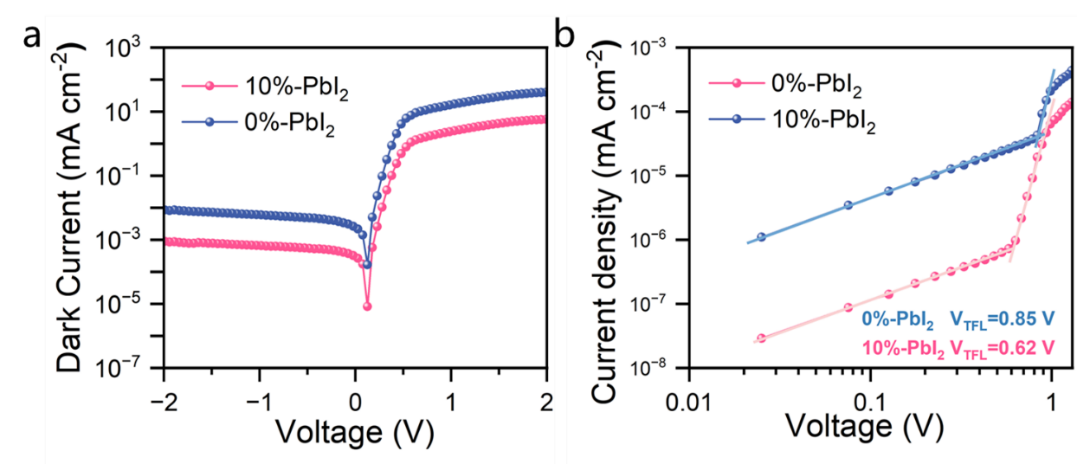


**Fig. S22** (a) Dark currents and (b) SCLC measurement of 0%-PbI_2_ and 10%-PbI_2_ based small-area devices (FTO/SnO_2_/perovskite/Spiro-OMeTAD/Au)


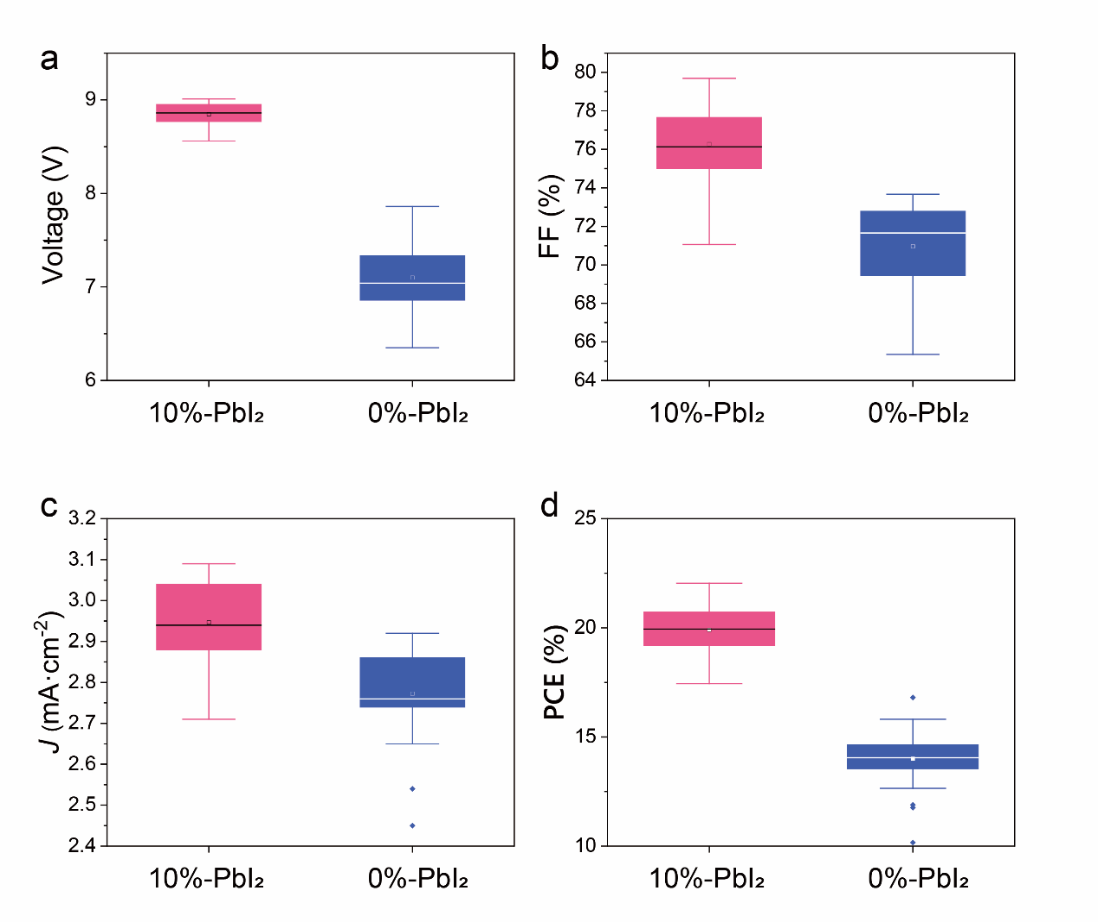


**Fig. S23** Performance of 25 individual 0%-PbI_2_ and 10%-PbI_2_ 18 cm^2^-PSMs devices under NIRA process


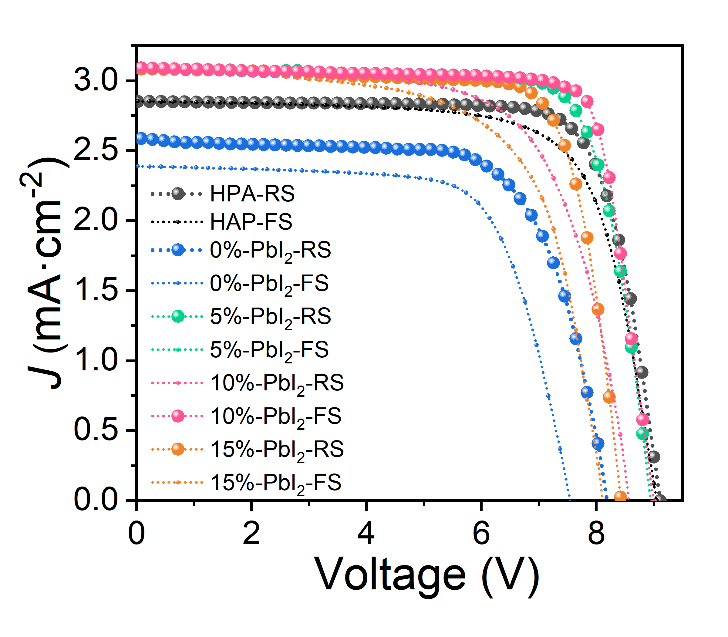


**Fig. S24** The *J-V* curves of perovskite modules prepared using HPA as well as those fabricated with 0%, 5%, and 15%-NIRA treatments

**Table S1** Fitted results of TRPL curves of Perovskite films under HPA and NIRA for 16 to 22s. TRPL curves were fitted using a bi-exponential decay equation of I(t) = I_0_ + A_1_exp(-t/τ_1_) + A_2_exp(-t/τ_2_)

|  | **τ_1_ (ns)** | **A2** | **τ_2_ (ns)** | **A2** | **τ_2_ (aver)** |
| --- | --- | --- | --- | --- | --- |
| **NIRA-16s** | 23.00 | 62.82 | 120.58 | 39.23 | 97.74 |
| **NIRA-18s** | 28.46 | 11.84 | 155.28 | 69.02 | 151.42 |
| **NIRA-20s** | 21.18 | 14.43 | 292.25 | 76.48 | 288.59 |
| **NIRA-22s** | 50.00 | 11.11 | 237.53 | 72.40 | 231.66 |
| **HAP-60min** | 42.00 | 18.01 | 420.52 | 74.70 | 411.61 |

**Table S2** The I/Pb ratios for the 0%-PbI_2_ and 10%-PbI_2_ samples

|  | **I** | **Pb** | **I/Pb rate** |
| --- | --- | --- | --- |
| **0%-PbI_2_** | 62311 | 17909 | 3.48 |
| **10%-PbI_2_** | 72868 | 22771 | 3.20 |

**Table S3** Performance of HPA-PSMs and NIRA-PSMs

|  | **Voltage/V** | ***J* /mA cm^-2^** | **FF%** | **RS%** |
| --- | --- | --- | --- | --- |
| HPA-RS | 1.15 | 25.51 | 81.87 | 24.02 |
| HAP-FS | 1.14 | 25.39 | 77.89 | 22.76 |
| 10%-PbI_2_-NIRA-RS | 1.17 | 25.71 | 80.73 | 24.33 |
| 10%-PbI_2_-NIRA-FS | 1.17 | 25.46 | 79.14 | 23.57 |

**Table S4** The champion PCE of 18 cm^2^-PSMs (6*6 cm) and 56 cm^2^-PSMs (10*10 cm) under NIRA process

|  | **Voltage/V** | ***J* /mA cm^-2^** | **FF%** | **RS%** | **FS%** |
| --- | --- | --- | --- | --- | --- |
| 18 cm^2^ | 9.007 | 3.092 | 79.14 | 22.03 | 18.84 |
| 56 cm^2^ | 14.86 | 1.713 | 79.27 | 20.18 | 17.28 |

**Table S5** Photovoltaic performances of PSMs based on HAP and NIRA treatment

|  | ***V*_oc_**  **(V)** | ***J***  **(mA cm^-2^)** | **FF**  **(%)** | **RS**  **(%)** | **FS**  **(%)** |
| --- | --- | --- | --- | --- | --- |
| HAP-0% | 9.109 | 2.851 | 77.27 | 20.07 | 18.59 |
| NIR-0% | 8.179 | 2.586 | 69.25 | 14.65 | 12.76 |
| NIR-5% | 8.933 | 3.091 | 77.56 | 21.42 | 17.84 |
| NIR-10% | 9.017 | 3.092 | 79.14 | 22.03 | 18.84 |
| NIR-15% | 8.409 | 3.088 | 76.85 | 19.96 | 16.48 |

**Table S6** High efficiency records of perovskite solar cells fabricated via blade coating

|  | **PSCs structure** | **PSCs PCE**  **(%)** | **PSMs**  **(AA cm^2^)** | **PSMs PCE (%)** | **Annealing condition** | **Year** |
| --- | --- | --- | --- | --- | --- | --- |
| 1 | ITO/SnO_2_/Perovskite/Spiro-OMeTAD/Au | 23.19 | 2.7 | 18.95 | 120^o^C  60 min | 2022 [S1] |
| 2 | ITO/PTAA/2-PACz/ Perovskite/C_60_/BCP/Cu | 24.31 | / | / | 120^o^C  20 min | 2023 [S2] |
| 3 | ITO/PTAA/Perovskite/PC_70_BM/BCP/Ag | 24.04 | / | / | 105^o^C  15 min | 2023 [S3] |
| 4 | FTO/TiO_2_/Perovskite/Spiro-OMeTAD/Au. | 24.00 | 1 | 18.37 | 150^o^C  15 min | 2023 [S4] |
| 5 | ITO/PTAA/Perovskite /BCP/C_60_/Cu | 23.32 | 1 | 22.23 | 100^o^C  60 min | 2023 [S5] |
| 6 | ITO/SnO_2_/Perovskite/Spiro-OMeTAD/Ag | 23.14 | 10.93 | 17.51 | 150^o^C  20 min | 2023 [S6] |
| 7 | ITO/HTL/Perovskite/C_60_/BCP/copper (Cu) | 25.21  (spin coating) | 26.9 | 21.8 | 100 °C 1 h & 150 °C 10 min. | 2023 [S7] |
| 8 | ITO/PTAA/2-PACz /Perovskite/C_60_/BCP/Cu | 24.16 | / | / | 150^o^C  30 min | 2024 [S8] |
| 9 | ITO/SnO_2_ /Perovskite/Spiro-OMeTAD/Ag | 24.67 | 11.35 | 19.45 | 150^o^C  20 min | 2024 [S9] |
| 10 | FTO/SnO_2_/Perovskite/Spiro-OMeTAD/Au | 24.32 | 15.64 | 21.90 | 150^o^C  10 mi | 2024 [S10] |
| 11 | FTO/SnO_2_/Perovskite/Spiro-OMeTAD/Au | 23.25 | 12.4 | 23.09 | 135°C  20 min | 2024 [S11] |
| 12 | FTO/SnO_2_/Perovskite/Spiro-OMeTAD/Au | 24.33 | 18.0 | 22.03 | **NIRA**  **20 s** | This work |

**Table S7** Photovoltaic performances of PSMs before and after aging test at 25±10 ^o^C and 60±10% RH

|  | **Ageing duration (h)** | **Voltage/V** | ***J* /mA cm^-2^** | **FF%** | **PCE%** |
| --- | --- | --- | --- | --- | --- |
| 10%-PbI_2_ | 0 | 9.007 | 3.092 | 79.14 | 22.03 |
|  | 1000 | 8.772 | 3.024 | 75.32 | 19.98 |
| 0%-PbI_2_ | 0 | 8.179 | 2.586 | 69.25 | 14.65 |
|  | 180 | 7.324 | 2.380 | 54.72 | 9.54 |

**Table S8** Photovoltaic performances of PSMs before and after aging test at 85^o^C and 85% RH

|  | **Ageing duration (h)** | **Voltage/V** | ***J* /mA cm^-2^** | **FF%** | **PCE%** |
| --- | --- | --- | --- | --- | --- |
| 10%-PbI_2_ | 0 | 7.933 | 2.847 | 68.63 | 16.04 |
|  | 1000 | 7.836 | 2.804 | 66.27 | 14.56 |
| 0%-PbI_2_ | 0 | 6.421 | 2.686 | 60.42 | 10.42 |
|  | 180 | 6.274 | 2.143 | 54.72 | 7.36 |

**Supplementary References**

1. Q. Liang, K. Liu, M. Sun, Z. Ren, P.W.K. Fong et al., Manipulating crystallization kinetics in high-performance blade-coated perovskite solar cells *via* cosolvent-assisted phase transition. Adv. Mater. **34**(16), e2200276 (2022). <https://doi.org/10.1002/adma.202200276>
2. W. Feng, J. Tao, G. Liu, G. Yang, J.-X. Zhong et al., Near-stoichiometric and homogenized perovskite films for solar cells with minimized performance variation. Angew. Chem. Int. Ed. **62**(17), e202300265 (2023). <https://doi.org/10.1002/anie.202300265>
3. J. Zhuang, C. Liu, B. Kang, H. Cheng, M. Xiao et al., Rapid surface reconstruction in air-processed perovskite solar cells by blade coating. Adv. Mater. **36**(6), 2309869 (2024). <https://doi.org/10.1002/adma.202309869>
4. Y. Du, Q. Tian, S. Wang, L. Yin, C. Ma et al., Crystallization control based on the regulation of solvent–perovskite coordination for high-performance ambient printable FAPbI3 perovskite solar cells. Adv. Mater. **36**(9), 2307583 (2024). <https://doi.org/10.1002/adma.202307583>
5. H. Zhu, B. Shao, J. Yin, Z. Shen, L. Wang et al., Retarding ion migration for stable blade-coated inverted perovskite solar cells. Adv. Mater. **36**(9), e2306466 (2024). <https://doi.org/10.1002/adma.202306466>
6. J. Chang, E. Feng, H. Li, Y. Ding, C. Long et al., Crystallization and orientation modulation enable highly efficient doctor-bladed perovskite solar cells. Nanomicro Lett. **15**(1), 164 (2023). <https://doi.org/10.1007/s40820-023-01138-x>
7. B. Ding, Y. Ding, J. Peng, J. Romano-deGea, L.E.K. Frederiksen et al., Dopant-additive synergism enhances perovskite solar modules. Nature **628**(8007), 299–305 (2024). <https://doi.org/10.1038/s41586-024-07228-z>
8. W. Feng, X. Liu, G. Liu, G. Yang, Y. Fang et al., Blade-coating (100)-oriented α-FAPbI_3_ perovskite films *via* crystal surface energy regulation for efficient and stable inverted perovskite photovoltaics. Angew. Chem. Int. Ed. **63**(39), e202403196 (2024). <https://doi.org/10.1002/anie.202403196>
9. J. Chang, E. Feng, X. Feng, H. Li, Y. Ding et al., Bridging buried interface enable 24.67%-efficiency doctor-bladed perovskite solar cells in ambient condition. Nano Res. **17**(9), 8068–8076 (2024). <https://doi.org/10.1007/s12274-024-6639-9>
10. L. Yuan, X. Chen, X. Guo, S. Huang, X. Wu et al., Volatile perovskite precursor ink enables window printing of phase-pure FAPbI_3_ perovskite solar cells and modules in ambient atmosphere. Angew. Chem. Int. Ed. **63**(7), e202316954 (2024). <https://doi.org/10.1002/anie.202316954>
11. C. Huang, S. Tan, B. Yu, Y. Li, J. Shi et al., *Meniscus*-modulated blade coating enables high-quality α-phase formamidinium lead triiodide crystals and efficient perovskite minimodules. Joule **8**(9), 2539–2553 (2024). <https://doi.org/10.1016/j.joule.2024.06.008>
